# Fluid balance control in critically ill patients: results from as-treated analyses of POINCARE-2 randomized trial

**DOI:** 10.1186/s13054-023-04701-5

**Published:** 2023-11-06

**Authors:** Adil Mansouri, Marie Buzzi, Sébastien Gibot, Claire Charpentier, Francis Schneider, Guillaume Louis, Hervé Outin, Alexandra Monnier, Jean-Pierre Quenot, Julio Badie, Laurent Argaud, Cédric Bruel, Marc Soudant, Nelly Agrinier, the POINCARE-2 group, Camille Alleyrat, Jean-Marc Virion, Pierre-Edouard Bollaert, Jérémie Lemarie, Ionel Alb, Pascal Welfringer, Rostane Gaci, Michel Bemer, Eric Delaveuve, Elsa Tahon, Pascal Andreu, Marie Labruyere, Jean-Baptiste Roudaut, Bruno Maire, Laurent Ziegler, Vincent Castelain, François Philippart, Marc Tran, Martin Cour, Marie Simon, Neven Stevic, Jann Hayon, Matthieu Jamme, Fernando Berdaguer, Hakim Slimani

**Affiliations:** 1 CHRU Nancy Hôpitaux de Brabois, INSERM, CIC, Epidémiologie Clinique, Université de Lorraine, 9 Allée du Morvan, 54000 Vandœuvre-lès-Nancy, Nancy, France; 2 APEMAC, Université de Lorraine, 54500 Nancy, France; 3 Service de Réanimation Médicale, CHRU Nancy, Université de Lorraine, 54000 Nancy, France; 4 Service d’Anesthésie Réanimation Chirurgicale, CHRU Nancy, Université de Lorraine, 54000 Nancy, France; 5 Service de Médecine Intensive-Réanimation, CHU Strasbourg, INSERM U 1121, Hôpital de Hautepierre, 67000 Strasbourg, France; 6 Service de Réanimation Polyvalente, CHR Metz-Thionville, 57000 Metz, France; 7 Service de Réanimation, CHI Poissy Saint-Germain, 78303 Poissy, France; 8 Service de Médecine Intensive-Réanimation Médicale, Nouvel Hôpital Civil, CHU Strasbourg, Université de Strasbourg, 67000 Strasbourg, France; 9 Service de Médecine Intensive-Réanimation, CHU Dijon-Bourgogne, 21000 Dijon, France; 10 Service de Réanimation Médicale, Hôpital Nord Franche-Comté, 90015 Belfort, France; 11 Service de Réanimation Médicale, Hospices Civils de Lyon, Hôpital Edouard Herriot, 69000 Lyon, France; 12 Service de Réanimation Polyvalente, Groupe Hospitalier Paris Saint-Joseph, 75000 Paris, France

**Keywords:** Critical care, Water–electrolyte balance, Clinical trial, Complex intervention, Instrumental variable

## Abstract

**Background:**

Intention-to-treat analyses of POINCARE-2 trial led to inconclusive results regarding the effect of a conservative fluid balance strategy on mortality in critically ill patients. The present as-treated analysis aimed to assess the effectiveness of actual exposure to POINCARE-2 strategy on 60-day mortality in critically ill patients.

**Methods:**

POINCARE‑2 was a stepped wedge randomized controlled trial. Eligible patients were ≥ 18 years old, under mechanical ventilation and had an expected length of stay in ICU > 24 h. POINCARE-2 strategy consisted of daily weighing over 14 days, and subsequent restriction of fluid intake, administration of diuretics, and/or ultrafiltration. We computed a score of exposure to the strategy based on deviations from the strategy algorithm. We considered patients with a score ≥ 75 as exposed to the strategy. We used logistic regression adjusted for confounders (ALR) or for an instrumental variable (IVLR). We handled missing data using multiple imputations.

**Results:**

A total of 1361 patients were included. Overall, 24.8% of patients in the control group and 69.4% of patients in the strategy group had a score of exposure ≥ 75. Exposure to the POINCARE-2 strategy was not associated with 60-day all-cause mortality (ALR: OR 1.2, 95% CI 0.85–1.55; IVLR: OR 1.0, 95% CI 0.76–1.33).

**Conclusion:**

Actual exposure to POINCARE-2 conservative strategy was not associated with reduced mortality in critically ill patients.

*Trial registration* POINCARE-2 trial is registered at ClinicalTrials.gov (NCT02765009). Registered 29 April 2016.

**Supplementary Information:**

The online version contains supplementary material available at 10.1186/s13054-023-04701-5.

## Background

Among critically ill patients in intensive care units (ICU), early and aggressive fluid resuscitation is indicated to tackle hemodynamic instability [1, 2]. However, after this early critical phase, excessive fluid intake can be deleterious by increasing intravascular pressure and vascular permeability, eventually leading to tissue edema [3].

Higher mortality was reported in critically ill patients with a positive fluid balance in various clinical settings, such as septic shock, acute respiratory distress syndrome (ARDS), cancers, and post-operative settings [4–7]. A meta-analysis of 11 randomized controlled trials included 2051 adults and children and showed that exposure to a strategy of conservative fluid management was associated with an increased number of mechanical ventilator-free days (MVFDs) and a reduced ICU length of stay, as compared to a liberal strategy or to standard of care. However, its effect on mortality remained uncertain [8].

POINCARE-2 stepped-wedge randomized controlled trial aimed to assess the effectiveness of a conservative strategy on 60-day all-cause mortality in a broad population of critically ill patients [9]. Intention-to-treat (ITT) analysis of POINCARE-2 trial led to inconclusive results, with a 60-day mortality of 30.5% (95% confidence interval [CI] 26.2–34.8) in the intervention group vs. 33.9% (95% CI 29.6–38.2) in the control group (*p* = 0.26) [10]. The strategy under scrutiny consisted of daily weighing over 14 days after admission to the ICU, and subsequent restriction of fluid intake, administration of diuretics, and/or ultrafiltration in case of renal replacement therapy (RRT) [9]. Accordingly, patients from the control group were likely to receive some of the strategy components as part of standard of care, despite the stepped-wedge design of the trial, which would result in so-called (but unavoidable) contamination and bias the results of ITT analyses towards the null [11]. Consequently, relying on ITT analyses only might lead to discredit a strategy that might otherwise prove effective if actual exposure to this strategy was taken into account.

The present as-treated analysis aimed to assess the effectiveness of actual exposure to POINCARE-2 strategy on 60-day mortality in critically ill patients.

## Methods

### Design and setting

The POids INtensive CARE 2 (POINCARE-2) trial was a stepped wedge cluster randomized controlled trial implemented in 12 French ICUs [9]. This trial aimed to assess the effectiveness of a fluid-balance control strategy on 60-day all-cause mortality in critically ill patients. As recommended [12], the main analysis was conducted on an intention-to-treat basis. However, to assess the actual magnitude of the strategy effect, as-treated analyses were also planned in the trial protocol as secondary analyses [9].

Critically ill patients who were admitted to one of the 12 recruiting ICUs were allocated either to the control group (and received standard of care during the control period) or to the strategy group (and received the POINCARE-2 strategy during the intervention period). They were followed up for 1 year.

Additional file 1: Fig. S1 describes the POINCARE-2 strategy. Briefly, this strategy relied on daily weighing from Day2 to Day14 after admission and subsequent daily decision to restrict salt and water for all infusion volumes (i.e., IV treatments and infusions allowing venous permeability), to administer diuretics and/or albumin, and/or to use ultrafiltration, in case of excessive weight gain.

### Population and sampling

Eligible patients were ≥ 18 years old, under mechanical ventilation (through endotracheal intubation), admitted to one of the 12 participating ICUs between 48 and 72 h prior to inclusion, and with an expected length of stay after inclusion of > 24 h.

Main exclusion criteria were clinical condition or unavailability of bedside scale impeding weight assessment, multiple trauma, history of ICU stay > 24 h immediately preceding the index ICU admission, pregnancy, expected withdrawal of life-sustaining therapy < 7 days after admission, patient refusal to personal data collection (and/or use), history of ICU stay in one of the 12 recruiting ICUs during the study period, and patients under guardianship.

### Assessment of exposure to the strategy

To assess exposure of patients to the strategy under scrutiny, we developed a dedicated score. This score was based on the amount of deviation from the algorithm on which the strategy relied (Additional file 1: Fig. S1), during the time over which it was supposed to be delivered (i.e., from Day2 to Day14 or the end of ICU stay which ever came first), for each patient, regardless of their group (strategy or control).

Additional file 1: Fig. S2 presents the scoring method. For each day of hospitalization in the recruiting ICU between Day2 and Day14 after admission, we incremented a counter of deviation from the algorithm at each step of the strategy, resulting in a crude score of deviation. To minimize the impact of missing data, we only considered the core components of the strategy (i.e., daily weighing, subsequent prescription of water and salt restriction, and administration of diuretics or ultrafiltration). We then normalized the crude score by dividing it by the length of ICU stay (or by 14 when length of stay was > 14 days) and transformed it to make it vary from 0 (i.e., POINCARE-2 strategy not administered at all) to 100 (i.e., POINCARE-2 strategy optimally administered as planned in the algorithm). We finally weighed it by the percentage of patient weighing performed between Day2 and Day14. To assess the validity of this score, we submitted 10 randomly selected patient files to 11 intensive care specialists. We stratified the random sampling on quintiles of score of exposure to the strategy, and randomly selected two patients per quintile. Each expert ranked the 10 patients according to his/her assessment of the compliance with the strategy, from 1 (best compliance with the strategy) to 10 (poorer compliance with the strategy). Experts were blinded to patient group, recruiting ICU and hospital, and to the score of exposure to the strategy. We found an agreement between experts for a computed score above 74.4. Accordingly, we further considered patients as actually exposed to the strategy if their score was ≥ 75, and as unexposed otherwise.

### Outcomes

The main outcome was vital status at Day 60 (alive vs deceased). Secondary outcomes included mechanical ventilator-free days (MVFDs) and vasopressor-free days (VFDs), defined as the cumulative number of days alive with no mechanical ventilation (or no prescription of vasopressor, respectively) between Day0 and Day28; renal replacement therapy-free days (RRTFDs), defined as the cumulative number of days alive with no renal replacement therapy between Day0 and Day60; occurrence of at least one unexpected harmful event (arterial hypotension, i.e. arterial systolic pressure < 90 mmHg, between Day2 and Day14; hypernatremia, i.e. serum sodium level > 155 mmol/L, between Day2 and Day14; hypokalemia, i.e. serum potassium level < 2.8 mmol/L, between Day2 and Day14; or acute ischemic events, i.e. myocardial infarction and/or patent mesenteric ischemia, between Day3 and discharge); and renal damage, defined by a worsening in the RIFLE criteria between Day3 and Day14, as compared to the higher RIFLE criteria during the first two days of hospitalization [13].

### Statistical analysis

#### Descriptive analyses

We first described the distribution of the score of exposure to the strategy in our sample, overall and by group. We then described patients’ characteristics at admission, overall, and stratified on (1) the quartile of score of exposure to the strategy, and (2) both the ICU and the group, using mean (standard deviation [SD]) or median (interquartile range [IQR]) for continuous variables, and counts (percentages) for categorical variables.

Then, we described survival time, vital status at Day 60, and total number (%) of events for each secondary outcome, overall and stratified on (1) the quartile of the score of exposure to the strategy, and (2) both the ICU and the group.

#### Main outcome

To assess the effect of actual exposure to the POINCARE-2 strategy on vital status at Day60, two different analyses were carried out: (1) a “naive” logistic regression model adjusted for potential confounders (model 1); and (2) a logistic regression model with the addition of an instrumental variable using the two-stage residual inclusion (2SRI) method [14] (model 2). Instrumental variable are useful to take into account both measured and unmeasured confounders [15]. To account for the stepped-wedge design of the trial, we adjusted the baseline model (model 0) for ICU and period.

To identify potential confounders in the association between exposure to the strategy and outcomes, we compared patients’ characteristics at admission according to the exposure to the strategy, using logistic regression models adjusted for both ICU and group. Patients’ characteristics that were associated with both the outcome of interest and the score of exposure to POINCARE-2 strategy with a *p* value of < 0.2 were further defined as potential confounders, and entered as dependent variables in adjusted analyses.

Hypotheses of log-linearity and absence of multiple colinearity were verified before implementation of the regression models, using the Hosmer–Lemeshow test [16] and calculation of (Generalized) Variance Inflation Factors, respectively [17].

In order to meet the assumptions of relevance, independence, and exclusion restriction, we chose the group (strategy *vs*. control) as an instrumental variable in model 2. We verified the relevance condition using a logistic regression explaining exposure to the strategy and the corresponding Wald test using F-value that was considered satisfactory above 10. We used a robust ridge regression estimation to account for the strong multiple collinearity between ICU, period, and group, inherent to the stepped-wedge design. We handled standard errors using bootstrap iterations.

Finally, we used the Durbin-Wu-Hausman [18] test to assess the relevance of the instrumental variable method, as compared with the naive logistic regression model.

We handled missing data using multiple imputation (MI), with the number of imputation datasets defined according to the rule of the percentage of missing observations [19]. We used the MI Boot method to combine bootstrap and multiple imputations [20].

#### Subgroup analyses

We added an interaction term to the naive logistic regression model (model 1) to test for a possible subgroup effect of the POINCARE-2 strategy according to the main cause of admission (septic shock, Acute Respiratory Distress Syndrome (ADRS), Central Nervous System (CNS) injury, or other).

#### Sensitivity analyses

We conducted sensitivity analyses for the main outcome in the subsample of complete cases.

#### Secondary outcomes

We used zero-inflated negative binomial (or zero-inflated Poisson) mixed models to assess the effect of exposure to the POINCARE-2 strategy on the cumulative number of MVFDs and VFDs. Logistic regression models adjusted for potential confounders were computed to examine the effect of the strategy on the occurrence of at least one unexpected harmful event or renal damage. We conducted all secondary analyses on complete cases only. We excluded from the RRTFDs analysis patients who were already at the worst stage of the RIFLE classification (i.e., “end stage renal disease”) at Day2.

We used SAS 9.4 (SAS Institute, Inc) and R v4.0.3, with a level of significance set at 0.05.

### Ethics

Because the POINCARE-2 strategy focused on health care organization, written informed consent was waived in accordance with French law (Bill number 2012–300 on March 5, 2012 about research involving humans). *Comité de Protection des Personnes Est III*, Grand-Est, North-East France, has reviewed and approved POINCARE-2 trial (ID-RCB: 2015-A00662-47). The trial is registered at ClinicalTrials.gov (NCT 02,765,009).

## Results

### Patients’ characteristics at admission

**Fig. 1:**
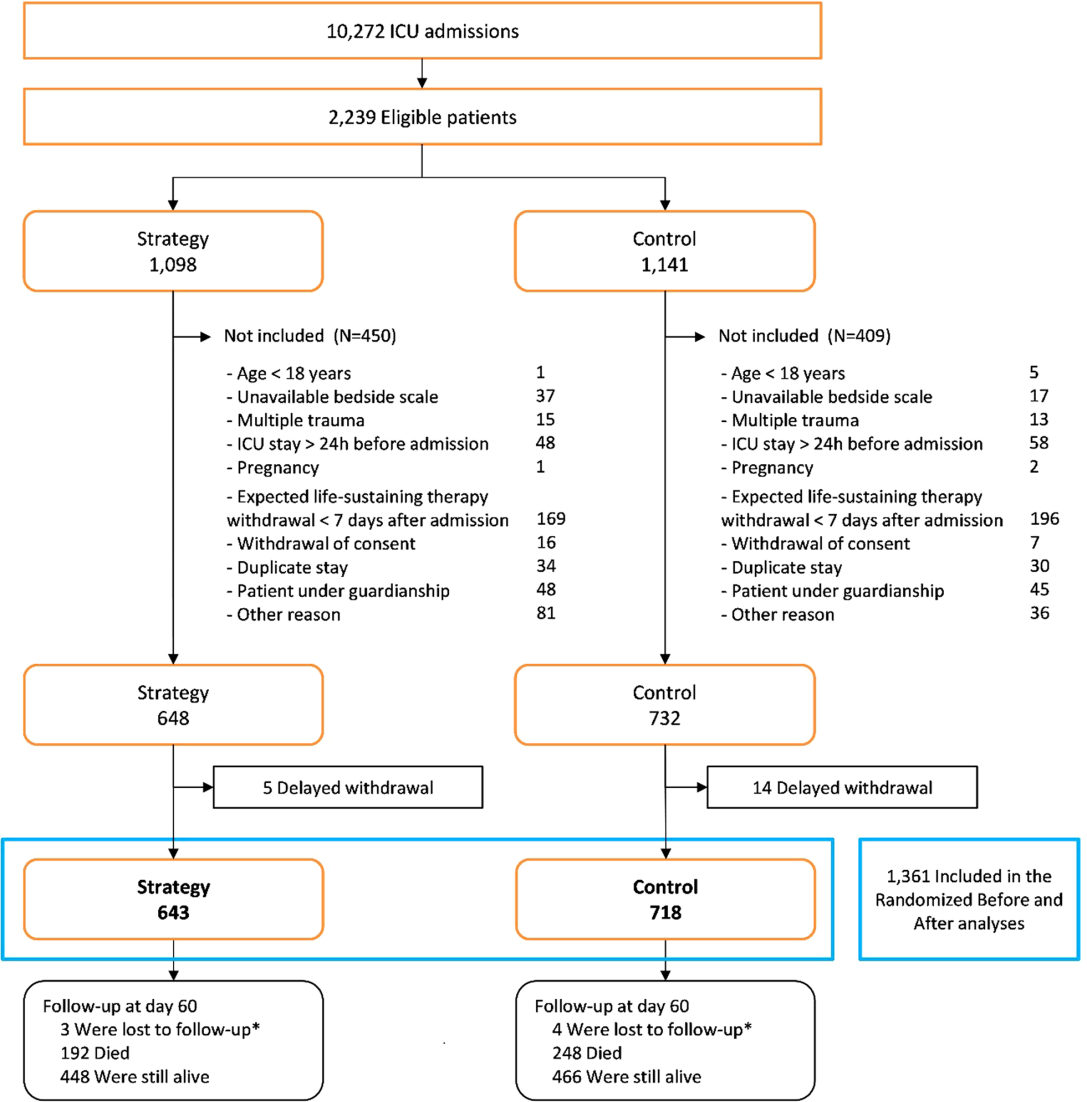
Flowchart of critically ill patients recruited and followed up in the POINCARE-2 trial

A total of 1361 patients were included in the present study, i.e. 718 in the control group and 643 in the strategy group (Fig. 1) [10]. Thirty patients were already at the worst stage of the RIFLE classification on Day2 and were further excluded for the RRTFDs analysis. We described patients’ characteristics at admission in Additional file 1: Tables S1 and S2. Mean (SD) age was 64.4 (14.6) years. Body mass index (BMI) and main cause of admission varied across ICUs, as did the presence of coexisting conditions such as heart failure or chronic kidney disease (Additional file 1: Table S2). Complete cases represented a subsample of 977 patients. Description of missing values is presented in Additional file 1: Table S3.

### Exposure to the POINCARE-2 strategy

Mean (SD) score of exposure to the strategy was 80.5 (19.3) in the strategy group vs. 55.2 (24.6) in the control group (overall, 67.1 (25.6)). Overall, 446 (69.4%) patients in the strategy group vs. 178 patients (24.8%) in the control group, had a score of exposure to the strategy ≥ 75 and were further considered as exposed to the strategy. Accordingly, despite higher scores of exposure, implementation of the strategy was not optimal in the strategy group, and a significant proportion of patients from the control group received at least some part of the strategy.

Implementation of the various components of the POINCARE-2 strategy are described in Table 1. The average daily fluid intake from Day2 to Day14 did not vary with exposure to the strategy (*p* = 0.249). Total dose of diuretics from Day0 to Day14 differed between groups, with the highest dose in the [75;100] class of exposure to the strategy.

**Table 1:** Characteristics of patients’ stays and implementation of the POINCARE-2 strategy

	Score of exposure to the strategy					Total N = 1361	Tests
	[0;25[	[25;50[	[50;75[	[75;100]			
	N = 74	N = 288	N = 375	N = 624			
Duration of stays in icu (days)							Kruskal–Wallis: 0.020
N	73	284	369	610		1336	
Mean (std)	14.1 (10.84)	15.0 (10.48)	13.5 (9.83)	15.9 (11.76)		15.0 (10.98)	
Min–median–max	2–11.0–58	4–12.0–58	3–11.0–56	2–12.0–60		2–11.0–60	
2	1 (1.4%)	0 (0.0%)	0 (0.0%)	1 (0.2%)		2 (0.1%)	
3	7 (9.5%)	0 (0.0%)	6 (1.6%)	7 (1.1%)		20 (1.5%)	
4	5 (6.8%)	22 (7.6%)	23 (6.1%)	15 (2.4%)		65 (4.8%)	
5	0 (0.0%)	18 (6.3%)	19 (5.1%)	50 (8.0%)		87 (6.4%)	
6	7 (9.5%)	20 (6.9%)	22 (5.9%)	42 (6.7%)		91 (6.7%)	
7	3 (4.1%)	10 (3.5%)	42 (11.2%)	35 (5.6%)		90 (6.6%)	
8	5 (6.8%)	13 (4.5%)	30 (8.0%)	32 (5.1%)		80 (5.9%)	
9	2 (2.7%)	16 (5.6%)	19 (5.1%)	44 (7.1%)		81 (6.0%)	
10	5 (6.8%)	24 (8.3%)	23 (6.1%)	34 (5.4%)		86 (6.3%)	
11	2 (2.7%)	18 (6.3%)	22 (5.9%)	37 (5.9%)		79 (5.8%)	
12	3 (4.1%)	10 (3.5%)	16 (4.3%)	23 (3.7%)		52 (3.8%)	
13	3 (4.1%)	10 (3.5%)	22 (5.9%)	28 (4.5%)		63 (4.6%)	
14 or more	31 (41.9%)	127 (44.1%)	131 (34.9%)	276 (44.2%)		565 (41.5%)	
Extra-renal purification during d0-14	8 (10.8%)	37 (12.8%)	64 (17.1%)	180 (28.8%)		289 (21.2%)	Chi-2: < 0.001
Total ultrafiltration from day 0 to day 14 (ml)							
N	5	29	54	155		243	
Mean (std)	941.8 (549.09)	5900.4 (7994.25)	7621.5 (7286.60)	12098.5 (10496.76)		10134.4 (9844.67)	
Min–median–max	250–1000.0–1740	120–4200.0–39976	28–4866.0–30033	50–9824.0–56181		28–7156.0–56181	
Average daily fluid intake from day 0 to day 14 (ml)							F-test: 0.249
N	74	288	375	624		1361	
Mean (std)	2297.7 (723.21)	2469.1 (871.77)	2478.4 (858.66)	2404.2 (860.61)		2432.6 (855.99)	
Min–median–max	254–2189.9–4487	377–2345.1–7233	239–2420.3–5411	469–2292.4–5913		239–2328.3–7233	
Total dose of albumin 20% from day 0 to day 14 (ml)							Kruskal–Wallis: 0.016
N	12	48	51	91		202	
Mean (std)	583.3 (404.15)	471.9 (372.59)	672.5 (643.14)	818.7 (803.73)		685.4 (673.15)	
Min–median–max	100–450.0–1500	100–300.0–1500	100–400.0–3700	100–600.0–5100		100–500.0–5100	
Total dose of diuretics from day 0 to day 14 (mg)							Kruskal–Wallis: < 0.001
N	45	160	263	485		953	
Mean (std)	408.4 (1208.74)	326.0 (515.01)	665.6 (1605.31)	770.7 (1414.45)		649.9 (1366.05)	
Min–median–max	10–120.0–8140	20–140.0–3500	10–210.0–12636	20–300.0–13432		10–220.0–13432	
Contraindications	65 (87.8%)	252 (87.5%)	349 (93.1%)	590 (94.6%)		1256 (92.3%)	Chi-2: 0.001
At least one fluid vascular loading (over d2-d14)	44 (59.5%)	171 (59.4%)	250 (66.7%)	392 (62.8%)		857 (63.0%)	Chi-2: 0.242
Arterial hypotension (at least one episode over d2-d14)	45 (60.8%)	204 (70.8%)	287 (76.5%)	488 (78.2%)		1024 (75.2%)	Chi-2: 0.002
At least one episode of hypernatremia > 155 mmol/l (over d2-d14)							Fisher's Exact: 0.024
Missing	1	0	2	0		3	
No	67 (90.5%)	284 (98.6%)	362 (96.5%)	600 (96.2%)		1313 (96.5%)	
Yes	6 (8.1%)	4 (1.4%)	11 (2.9%)	24 (3.8%)		45 (3.3%)	
At least one episode of hypokalemia < 2.8 mmol/l (over d2-d14)							Chi-2: 0.715
Missing	1	0	1	0		2	
No	67 (90.5%)	264 (91.7%)	351 (93.6%)	576 (92.3%)		1258 (92.4%)	
Yes	6 (8.1%)	24 (8.3%)	23 (6.1%)	48 (7.7%)		101 (7.4%)	
At least one episode of non-normal rifle (over d2-d14)	51 (68.9%)	173 (60.1%)	266 (70.9%)	503 (80.6%)		993 (73.0%)	Chi-2: < 0.001
Percentage of weighing done from day 2 to day 14							Kruskal–Wallis: < 0.001
N	74	288	375	624		1361	
Mean (std)	16.4 (16.16)	47.3 (17.16)	70.0 (13.90)	92.9 (8.35)		72.8 (25.69)	
Min–median–max	0–16.0–92	25–44.4–100	50–66.7–100	75–100.0–100		0–77.8–100	

Patients exposed to POINCARE-2 strategy were more likely to be older (*p* < 0.001), to have a higher McCabe score at admission (*p* = 0.016), and to have a cancer (*p* = 0.031) or a chronic kidney disease under RRT (*p* = 0.008) than patients less exposed to the strategy (Additional file 1: Table S1).

### Mortality and safety during follow-up

Vital status at Day 60 and safety outcomes are described in Table 2. During an overall median (min–max) follow-up of 11 (2–60) days, a total of 440 patients (32.3%) died, i.e. 39.2% (28.8%, 30.1%, and 34.5%) of patients with a score of exposure to POINCARE-2 strategy comprised between [0–25[([25–50[, [50–75[, and [75–100], respectively). Seven patients were lost to follow-up.

**Table 2:** Occurrence of primary and secondary outcomes

	Score of exposure to the strategy				Total N = 1361	
	[0;25[	[25;50[	[50;75[	[75;100]		
	N = 74	N = 288	N = 375	N = 624		
Primary outcome						
Vital status at day 60						
Lost of follow-up	0	1	4	2	7	
Alive	45 (60.8%)	204 (70.8%)	258 (68.8%)	407 (65.2%)	914 (67.2%)	
Deceased	29 (39.2%)	83 (28.8%)	113 (30.1%)	215 (34.5%)	440 (32.3%)	
Survival time (days)—truncated at Day 60						
N	74	288	375	624	1361	
Mean (std)	43.5 (22.28)	48.3 (19.85)	47.0 (20.82)	46.2 (20.36)	46.7 (20.50)	
Min–median–max	3–60.0–60	4–60.0–60	3–60.0–60	2–60.0–60	2–60.0–60	
Secondary outcomes						
Effectiveness						
Number of days without mechanical ventilation during Day0 – Day28						
N	74	288	375	624	1361	
Mean (std)	8.4 (8.90)	10.2 (8.26)	9.9 (8.61)	9.4 (8.54)	9.7 (8.52)	
Min–median–max	0–5.0–26	0–10.0–26	0–9.0–28	0–8.0–27	0–9.0–28	
Number of days without vasopressor during Day0 – Day28						
N	74	288	375	624	1361	
Mean (std)	15.0 (10.07)	17.0 (9.59)	14.8 (9.48)	15.1 (9.46)	15.4 (9.55)	
Min–median–max	0–14.5–28	0–20.0–28	0–14.0–28	0–16.0–28	0–16.0–28	
Number of days without RRT (#) during Day0 – Day60						
N	74	288	375	624	1361	
Mean (std)	27.3 (20.50)	30.0 (19.90)	26.7 (19.57)	27.2 (20.31)	27.7 (20.05)	
Min–median–max	0–21.5–60	0–27.0–60	0–21.0–60	0–21.5–60	0–22.0–60	
Vital status at Day 28						
Missing	0	0	1	2	3	
Alive	51 (68.9%)	225 (78.1%)	279 (74.4%)	458 (73.4%)	1013 (74.4%)	
Deceased	23 (31.1%)	63 (21.9%)	95 (25.3%)	164 (26.3%)	345 (25.3%)	
In-hospital mortality at Day 60						
Missing	1	0	0	3	4	
No	46 (62.2%)	209 (72.6%)	266 (70.9%)	414 (66.3%)	935 (68.7%)	
Yes	27 (36.5%)	79 (27.4%)	109 (29.1%)	207 (33.2%)	422 (31.0%)	
Safety outcomes	47 (63.5%)	207 (71.9%)	293 (78.1%)	495 (79.3%)	1042 (76.6%)	
Arterial hypotension (at least one episode over D2-D14)	45 (60.8%)	204 (70.8%)	287 (76.5%)	488 (78.2%)	1024 (75.2%)	
At least one episode of hypernatremia > 155 mmol/L (over D2-D14)						
Missing	1	0	2	0	3	
No	67 (90.5%)	284 (98.6%)	362 (96.5%)	600 (96.2%)	1313 (96.5%)	
Yes	6 (8.1%)	4 (1.4%)	11 (2.9%)	24 (3.8%)	45 (3.3%)	
At least one episode of hypokalemia < 2.8 mmol/L (over D2-D14)						
Missing	1	0	1	0	2	
No	67 (90.5%)	264 (91.7%)	351 (93.6%)	576 (92.3%)	1258 (92.4%)	
Yes	6 (8.1%)	24 (8.3%)	23 (6.1%)	48 (7.7%)	101 (7.4%)	
Mesenteric ischemia or myocardial infarction	0 (0.0%)	0 (0.0%)	7 (1.9%)	7 (1.1%)	14 (1.0%)	
Mesenteric ischemia	0 (0.0%)	0 (0.0%)	2 (0.5%)	3 (0.5%)	5	(0.4%)
Myocardial infarction	0 (0.0%)	0 (0.0%)	5 (1.3%)	4 (0.6%)	9 (0.7%)	
Renal damage over D3-D14						
N/A	0	2	6	22	30	
No RIFLE after D2	4	0	1	3	8	
No D1-D2 RIFLE	0	1	1	4	6	
No	58 (78.4%)	230 (79.9%)	298 (79.5%)	467 (74.8%)	1053 (77.4%)	
Yes	12 (16.2%)	55 (19.1%)	69 (18.4%)	128 (20.5%)	264 (19.4%)	

(#) RRT: Renal-replacement therapy

Forty-five (3.3%) patients presented at least one episode of hypernatremia > 155 mmol/L, and 14 (1.0%) had an episode of mesenteric or myocardial infarction during follow-up.

### Effect of the exposure to the POINCARE-2 strategy on 60-day mortality

#### Results from the naive logistic regression

**Fig. 2:**
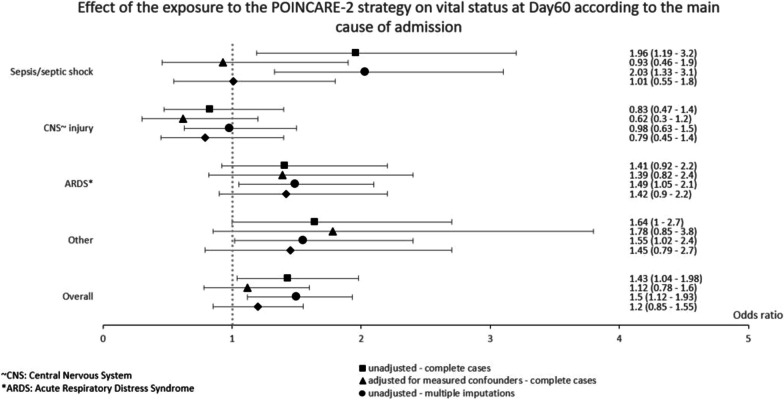
Effect of the exposure to the POINCARE-2 strategy on vital status at Day60 according to the main cause of admission

Results for the main outcome analyses are presented in Table 3 and Fig. 2. As compared with patients considered less exposed to the POINCARE-2 strategy, patients with a score of exposure ≥ 75 had a significantly higher mortality at Day60 (OR_model0_ 1.47, 95% CI 1.12–1.93, *p* = 0.005). Analyses on the complete cases subsample led to similar results (OR_model0_ 1.43, 95% CI 1.04–1.98, *p* = 0.029). Adjustment for potential confounders (i.e., age, presence or absence of heart failure or chronic kidney disease under RRT, McCabe Score at admission, SAPS II, main cause of admission, SOFA, and RRT at Day0) led to similar yet insignificant results (MI dataset: OR_model1_ 1.15, 95% CI 0.85–1.55, *p* = 0.35; complete case dataset: OR_model1_ 1.12, 95% CI 0.78–1.60, *p* = 0.547).

**Table 3:** Effect of the exposure to the POINCARE-2 strategy on vital status at Day60

	Complete cases^a^			Multiple imputation^b^		
	OR (Score > 75%)	95% CI	*p*	OR (Score > 75%)	95% CI	*p*
Unadjusted^c^	1.43	1.04–1.98	0.029	1.47	1.12–1.93	0.005
Adjusted for measured confounders^d^	1.12	0.78–1.60	0.547	1.15	0.85–1.55	0.356
Adjusted for measured and unmeasured confounders^e^	0.97	0.70–1.34	0.843	1.01	0.76–1.33	0.956

Models:

^c^Adjusted only on class variables: Center and Secular time

^d^adjusted on Center, Secular time, Age (years), Heart failure, Chronic kidney disease under RRT, McCabe Score at admission, SAPS II (*), Main cause of admission, SOFA ( ~), RRT (#) at day 0

^e^2SRI instrumental variable model adjusted on Center, Secular time, Age (years), Cirrhosis, Cancer, Immunodeficiency, Heart failure, Chronic respiratory failure, Chronic kidney disease under RRT, McCabe Score at admission, SAPS II (*), Main cause of admission, BMI (IOTF classification), Weight (kg), Serum bicarbonate (mmol/L), Serum potassium (mmol/L), SOFA ( ~), Serum creatinine (mg/dL), PaO^2^/FiO^2^ (mmHg), RRT (#) at day 0

Analyses:

^a^Observations with missing data are excluded (n = 977)

^b^Number of imputation datasets = 4 (n = 1361)

*SAPS: Simplified Acute Physiology Score

~ SOFA: Sequential Organ Failure Assessment

^#^RRT: Renal-replacement therapy

#### Results from instrumental variable analysis

Exposure to the POINCARE-2 strategy was not associated with 60-day mortality both in the MI dataset (OR_model2_ 1.01, 95% CI 0.76–1.33, *p* = 0.956) and in the complete case dataset (OR_model2_ 0.97, 95% CI 0.70–1.34, *p* = 0.843).

#### Results according to cause of admission: subgroup analyses

Results of the subgroup analyses are presented in Table 4. They showed that exposure to the strategy was associated with an increased 60-day mortality in case of ARDS, sepsis or septic shock, or other causes of admission. However, these results were no longer statistically significant when adjusting for potential confounders. Analyses on both MI and complete case datasets led to similar results.

**Table 4:** Effect of the exposure to the POINCARE-2 strategy on vital status at Day60 according to the main cause of admission

	Complete cases dataset						Imputed dataset					
	Bivariate			Multivariate			Bivariate			Multivariate		
	OR	95% CI	*p*	OR	95% CI	*p*	OR	95% CI	*p*	OR	95% CI	*p*
ARDS*	1.41	0.92–2.2	0.113	1.39	0.82–2.4	0.226	1.49	1.05–2.1	0.027	1.42	0.90–2.2	0.128
CNS ~ injury	0.83	0.47–1.4	0.524	0.62	0.30–1.2	0.174	0.98	0.63–1.5	0.922	0.79	0.45–1.4	0.408
Other	1.64	1.00–2.7	0.047	1.78	0.85–3.8	0.126	1.55	1.02–2.4	0.041	1.45	0.79–2.7	0.230
Sepsis/septic shock	1.96	1.19–3.2	0.007	0.93	0.46–1.9	0.829	2.03	1.33–3.1	0.001	1.01	0.55–1.8	0.980

*ARDS: Acute respiratory distress syndrome

~ CNS: Central nervous system

### Effect of exposure to the POINCARE-2 strategy on secondary outcomes

Results for secondary outcomes are presented in Additional file 1: Tables S4 and S5.

#### MVFDs

After adjustment for potential confounders, we found a negative association between exposure to the strategy and MVFDs, both on the imputed and on the complete case datasets (Additional file 1: Table S4).

#### VFDs

We found no significant association of the exposure to the POINCARE-2 strategy with the cumulative number of VFDs between Day0 and Day28 (Additional file 1: Table S4).

#### Unexpected harmful events

We found no significant association between exposure to the POINCARE-2 strategy and occurrence of at least one unexpected harmful event (i.e., arterial hypotension between Day2 and Day14, hypernatremia between Day2 and Day14, hypokalemia between Day2 and Day14, and acute ischemic events between Day3 and discharge) (Additional file 1: Table S5).

#### Renal damage

We found no significant association between exposure to the POINCARE-2 strategy and worsening in the RIFLE criteria between Day3 and Day14 (Additional file 1: Table S5).

## Discussion

Similarly to the intention-to-treat analyses [10], adjusted as-treated analyses showed no statistically significant association between exposure to the POINCARE-2 strategy and 60-day all-cause mortality in critically ill patients. The use of an instrumental variable to account for unmeasured confounders, as well as subgroup analyses according to main cause of admission, yielded similar findings. Taken together, these results make the effect of the POINCARE-2 intervention on mortality in a general population of critically ill patients unlikely.

These results are in line with previous findings. While the deleterious effect of fluid overload on mortality has been repeatedly reported in observational studies in critically ill patients [21], patients suffering from septic shock [22, 23], ARDS [24, 25], or traumatic brain injury [26], the beneficial effect of fluid balance control strategies has been more difficult to demonstrate. So far, trials failed to report any significant effect of a conservative strategy on mortality in patients with ARDS [27, 28] or septic shock [29, 30]. A recent meta-analysis led to similar conclusions with an overall RR 0.92; 95% CI 0.82–1.02 regarding the effect of conservative (vs. liberal) fluid balance control strategies on mortality [8]. However, conservative strategies have proven effective on other outcomes of interest, such as ICU length of stay and number of mechanical ventilator-free days in patients with ARDS [27, 28]. Although the effect of the POINCARE-2 strategy on such outcomes was not significant in intention-to-treat analyses [10], we found a significant decrease in the cumulative number of MVFDs in as-treated analyses, suggesting the POINCARE-2 intervention might actually be effective on this outcome in a broad population of critically-ill patients.

The highest evidence derived from randomized controlled trials (RCT) comes from intention-to-treat analyses of individual RCT [31]. However, in case of contamination [11], i.e., non-null exposure to the strategy under scrutiny in the control arm, or suboptimal exposure to the strategy in the strategy arm, results of intention-to-treat analyses are biased towards the null. As shown by our dedicated score of exposure to the strategy, we observed both contamination and suboptimal exposure to POINCARE-2 strategy in our trial. POINCARE-2 strategy relied on components, such as weighing, administration of diuretics or albumin, or RRT, that are commonly used as part of standard of care in ICU, and are indicated in many other clinical conditions than prevention of fluid overload [32–34]. It is thus very unlikely that patients from the control group of a RCT assessing the effectiveness of a fluid-balance control strategy would have no exposure to the strategy at all, even under optimal experimental conditions. In fact, only 5% of patients included in POINCARE-2 trial had a score of exposure to the strategy < 25. In the same way, as some clinical conditions contraindicate the administration of diuretics or fluid restriction, such as severe dehydration or established anuria [35], maximal exposure to the strategy could not be observed in all patients from the strategy group. In fact, in POINCARE-2 trial, although we observed a higher mean score of exposure to the strategy in the strategy group, not all patients from this group had a score of 100.

Having a closer look at the components of POINCARE-2 strategy, our results suggest that the proportion of daily weighing performed, as well as the total dose of diuretics, increased with an increasing exposure to the strategy. The daily average fluid intake, however, did not vary in the same way with the score of exposure to the strategy. This suggests that intensivists were more adherent to components of the strategy favoring fluid depletion than those favoring water and salt restriction to control fluid balance. They might also not be aware enough of how much creep fluid accounts for daily fluid intake. These findings are in line with the ones of a cross-sectional study among 524 critical care specialists showing that diuretics were the most frequently prescribed treatment to prevent fluid overload (66%). In this study, most intensivists reported using diuretics to treat fluid overload on at least 50% of days working in ICU [36].

Our study suffers from some limitations. First, although experts agreed to rank the patients with a score of exposure to the strategy > 75 as most exposed, their agreement on exposure to the strategy in patients with lower score of exposure was less consensual. Accordingly, the defined unexposed group mixed unexposed patients and somewhat exposed patients, without us being able to separate the last ones from the first ones. This might have biased our results towards the null. In addition, the highest 60-day mortality was observed both in the lowest ([0–25[) and in the highest ([75–100]) score of exposure categories, which either corroborates a potential bias on measurement of exposure to the strategy in the lowest score category or suggests that the effect of exposure to the strategy on mortality is J-shaped. This would mean that rather than discrediting conservative strategies, we might consider applying them using restrictive therapeutic targets and/or during a limited time window. Second, the participating ICUs were equipped with different weigh scales. Despite our recommendation to tare each scale before each weigh assessment, this might have led to measurement bias. Third, we did not size the trial sample to handle adjustment for multiple confounders or to use an instrumental variable without loss of power. This might have resulted in our inability to detect a significant effect of the strategy.

Our study further highlights the difficulty of demonstrating an effect of a conservative strategy in critical care practice, despite evidence of the deleterious effect of fluid overload. Given the dynamic nature of the POINCARE-2 strategy, and because of the constant need to adapt this strategy to patients’ clinical status, which varies over time, alternative statistical methods might be required in future studies. Especially, the target trial emulation approach, by simulating the various possible intervention regimens over time, could help compare the various treatment sequences and determine the most appropriate time to start fluid balance control after ICU admission [37].

Furthermore, the POINCARE-2 strategy relied on multiple components and involved multiple health professionals (i.e., nurses and assistant nurses for weighing and administration of prescribed medications, and intensivists for prescriptions). As thus, it can be considered as a complex intervention, as defined by the Medical Research Council [38]. Implementation and effects of such interventions may vary due to their interactions with the context of implementation [39]. In fact, patients’ characteristics (e.g., indications and contraindications of each of the strategy components), ICUs organizational characteristics (e.g., availability of weighing material, nursing staff size, inter professional conflicts…), and health professionals’ attitudes towards fluid balance control were likely to influence POINCARE-2 strategy implementation, and may have hindered optimal conditions to assess its effectiveness. As a consequence, only a complete process evaluation, as planned in POINCARE-2 trial protocol [9], could help untangle the interventional and contextual factors that may have influenced the actual implementation of the POINCARE-2 strategy [40].

## Conclusions

As-treated analyses did not show a significant effect of the POINCARE-2 fluid balance control strategy on mortality in a broad population of critically ill patients. Further research, such as target trial emulation or a complete process evaluation, might help understand the conditions required for conservative strategies to be effective.

### Supplementary Information

**Additional file 1:** Supplementary tables and figures.

## Data Availability

Due to restrictions pertaining to French laws, the datasets generated and/or analyzed during POINCARE-2 trial are not publicly available. However, data transfer agreement remains possible and data can be made available upon reasonable request to the corresponding author.

## Abbreviations

ARDS: Acute respiratory distress syndrome
CRA: Cluster randomized analyses
ICU: Intensive care unit
ITT: Intention-to-treat
MI: Multiple imputation
MVFD: Mechanical ventilator-free days
OR: Odds ratio
RBAA: Randomized before-and-after analyses
RCT: Randomized controlled trial
RR: Relative risk
RRTFD: Renal replacement therapy-free day
VFD: Vasopressor-free day
