# Supplementary material for: Fluid balance control in critically ill patients: results from as-treated analyses of POINCARE-2 randomized trial
Source: Crit Care. 2023 Nov 6;27:426. doi: 10.1186/s13054-023-04701-5 (PMC10626740; doi:10.1186/s13054-023-04701-5)

Electronic supplementary material

[Table S1. Patients characteristics at admission according to quartile of score of exposure to the POINCARE-2 strategy 2](#_Toc147760871)

[Table S2. Patients characteristics at admission according to ICU and intervention group (part 1 of 2) 6](#_Toc147760872)

[Table S2. Patients characteristics at admission according to ICU and intervention group (part 2 of 2) 12](#_Toc147760873)

[Table S3. Description of missing values 17](#_Toc147760874)

[Table S4. Effect of the exposure to the POINCARE-2 strategy on the cumulative number of MVFDs and VFDs 18](#_Toc147760875)

[Table S5. Effect of the exposure to the POINCARE-2 strategy on the occurrence of unexpected harmful events and renal damage 19](#_Toc147760876)

[Figure S1. POINCARE-2 strategy of fluid balance control in critically ill patients (reproduced from 9) 20](#_Toc147760877)

[Figure S2. Computation method for the score of exposure to the POINCARE-2 strategy 21](#_Toc147760878)

# Table S1. Patients characteristics at admission according to quartile of score of exposure to the POINCARE-2 strategy

|  | Score of exposure to the strategy | | | | | | | | Total | | Tests / p-values |
| --- | --- | --- | --- | --- | --- | --- | --- | --- | --- | --- | --- |
|  | [0;25[ | | [25;50[ | | [50;75[ | | [75;100] | |  |  |  |
|  | N=74 | | N=288 | | N=375 | | N=624 | | N=1361 | |  |
| **Age (years)** |  |  |  |  |  |  |  |  |  |  | Kruskal-Wallis : <0.001 |
| N | 74 | | 288 | | 375 | | 624 | | 1361 | |  |
| mean (std) | 63.1 (16.04) | | 61.6 (15.56) | | 64.1 (14.74) | | 66.0 (13.56) | | 64.4 (14.56) | |  |
| min - median - max | 21 - 66.0 - 94 | | 18 - 64.0 - 89 | | 18 - 66.0 - 92 | | 19 - 68.0 - 97 | | 18 - 66.0 - 97 | |  |
| **Male sex** | 50 | (67.6%) | 184 | (63.9%) | 245 | (65.3%) | 400 | (64.1%) | 879 | (64.6%) | Chi-2 : 0.918 |
| **Weight (kg)** |  |  |  |  |  |  |  |  |  |  | Kruskal-Wallis : <0.001 |
| N | 74 | | 288 | | 375 | | 624 | | 1361 | |  |
| mean (std) | 82.6 (23.57) | | 75.7 (19.02) | | 77.4 (23.85) | | 80.8 (21.28) | | 78.9 (21.80) | |  |
| min - median - max | 37 - 78.5 - 174 | | 39 - 73.5 - 156 | | 29 - 74.0 - 232 | | 38 - 79.0 - 172 | | 29 - 76.0 - 232 | |  |
| **Height (cm)** |  |  |  |  |  |  |  |  |  |  | F-test : 0.631 |
| N | 67 | | 277 | | 364 | | 600 | | 1308 | |  |
| mean (std) | 170.6 (9.91) | | 169.0 (9.36) | | 169.4 (9.86) | | 169.5 (9.61) | | 169.4 (9.64) | |  |
| min - median - max | 147 - 172.0 - 190 | | 135 - 170.0 - 195 | | 143 - 170.0 - 195 | | 142 - 170.0 - 200 | | 135 - 170.0 - 200 | |  |
| **Body mass index (kg/m²)** |  |  |  |  |  |  |  |  |  |  | Kruskal-Wallis : <0.001 |
| N | 67 | | 277 | | 364 | | 600 | | 1308 | |  |
| mean (std) | 28.4 (8.22) | | 26.6 (7.11) | | 27.0 (8.16) | | 28.1 (7.24) | | 27.5 (7.55) | |  |
| min - median - max | 16 - 26.2 - 64 | | 14 - 25.2 - 62 | | 14 - 25.2 - 76 | | 14 - 27.0 - 66 | | 14 - 26.0 - 76 | |  |
| missing | 7 |  | 11 |  | 11 |  | 24 |  | 53 |  | Chi-2 : 0.003 |
| Underweight | 3 | (4.1%) | 20 | (6.9%) | 33 | (8.8%) | 29 | (4.6%) | 85 | (6.2%) |  |
| Normal | 22 | (29.7%) | 111 | (38.5%) | 146 | (38.9%) | 191 | (30.6%) | 470 | (34.5%) |  |
| Overweight | 21 | (28.4%) | 84 | (29.2%) | 95 | (25.3%) | 182 | (29.2%) | 382 | (28.1%) |  |
| Obese | 21 | (28.4%) | 62 | (21.5%) | 90 | (24.0%) | 198 | (31.7%) | 371 | (27.3%) |  |
| **McCabe Score at admission** |  |  |  |  |  |  |  |  |  |  | Chi-2 : 0.016 |
| missing | 0 |  | 0 |  | 0 |  | 1 |  | 1 |  |  |
| Unknown | 3 |  | 27 |  | 25 |  | 36 |  | 91 |  |  |
| Non fatal | 48 | (64.9%) | 186 | (64.6%) | 246 | (65.6%) | 367 | (58.8%) | 847 | (62.2%) |  |
| Ultimately fatal | 21 | (28.4%) | 63 | (21.9%) | 91 | (24.3%) | 171 | (27.4%) | 346 | (25.4%) |  |
| Rapidly fatal | 2 | (2.7%) | 12 | (4.2%) | 13 | (3.5%) | 49 | (7.9%) | 76 | (5.6%) |  |
| **Coexisting conditions** |  |  |  |  |  |  |  |  |  |  |  |
| Cirrhosis | 2 | (2.7%) | 16 | (5.6%) | 26 | (6.9%) | 50 | (8.0%) | 94 | (6.9%) | Chi-2 : 0.268 |
| Cancer | 8 | (10.8%) | 32 | (11.1%) | 50 | (13.3%) | 112 | (17.9%) | 202 | (14.8%) | Chi-2 : 0.031 |
| Immunodeficiency | 11 | (14.9%) | 32 | (11.1%) | 36 | (9.6%) | 91 | (14.6%) | 170 | (12.5%) | Chi-2 : 0.102 |
| Heart failure | 5 | (6.8%) | 19 | (6.6%) | 30 | (8.0%) | 69 | (11.1%) | 123 | (9.0%) | Chi-2 : 0.088 |
| Diabetes mellitus | 18 | (24.3%) | 60 | (20.8%) | 101 | (26.9%) | 176 | (28.2%) | 355 | (26.1%) | Chi-2 : 0.155 |
| Chronic respiratory failure | 23 | (31.1%) | 50 | (17.4%) | 73 | (19.5%) | 145 | (23.2%) | 291 | (21.4%) | Chi-2 : 0.025 |
| Chronic kidney disease under RRT (#) | 0 | (0.0%) | 1 | (0.3%) | 5 | (1.3%) | 21 | (3.4%) | 27 | (2.0%) | Fisher Exact : 0.008 |
| **SAPS II (*)** |  |  |  |  |  |  |  |  |  |  | F-test : 0.002 |
| N | 74 | | 288 | | 372 | | 616 | | 1350 | |  |
| mean (std) | 54.4 (19.15) | | 56.2 (18.03) | | 58.9 (17.45) | | 60.3 (18.19) | | 58.7 (18.09) | |  |
| min - median - max | 12 - 53.0 - 105 | | 15 - 55.5 - 113 | | 21 - 58.0 - 115 | | 13 - 60.0 - 131 | | 12 - 58.0 - 131 | |  |
| **SOFA (~)** |  |  |  |  |  |  |  |  |  |  | F-test : 0.214 |
| N | 74 | | 288 | | 375 | | 624 | | 1361 | |  |
| mean (std) | 8.3 (3.39) | | 8.8 (3.79) | | 8.8 (3.69) | | 9.1 (3.86) | | 8.9 (3.78) | |  |
| min - median - max | 0 - 9.0 - 17 | | 0 - 9.0 - 18 | | 0 - 9.0 - 20 | | 0 - 9.0 - 19 | | 0 - 9.0 - 20 | |  |
| **Serum sodium (mmol/L)** |  |  |  |  |  |  |  |  |  |  | F-test : 0.229 |
| N | 69 | | 281 | | 366 | | 609 | | 1325 | |  |
| mean (std) | 136.2 (5.76) | | 137.8 (7.13) | | 138.0 (6.79) | | 137.9 (6.50) | | 137.8 (6.68) | |  |
| min - median - max | 120 - 137.0 - 148 | | 114 - 137.0 - 172 | | 106 - 138.0 - 162 | | 113 - 138.0 - 174 | | 106 - 138.0 - 174 | |  |
| **Serum potassium (mmol/L)** |  |  |  |  |  |  |  |  |  |  | Welch : 0.020 |
| N | 70 | | 282 | | 364 | | 604 | | 1320 | |  |
| mean (std) | 4.2 (0.87) | | 4.0 (0.85) | | 4.1 (0.88) | | 4.2 (1.02) | | 4.1 (0.94) | |  |
| min - median - max | 3 - 4.1 - 7 | | 2 - 3.9 - 8 | | 2 - 4.0 - 8 | | 1 - 4.1 - 9 | | 1 - 4.0 - 9 | |  |
| **Serum bicarbonate (mmol/L)** |  |  |  |  |  |  |  |  |  |  | F-test : 0.505 |
| N | 70 | | 272 | | 347 | | 588 | | 1277 | |  |
| mean (std) | 21.7 (5.58) | | 22.3 (6.77) | | 21.6 (7.41) | | 21.5 (7.26) | | 21.7 (7.12) | |  |
| min - median - max | 8 - 21.7 - 35 | | 3 - 22.0 - 46 | | 2 - 21.0 - 55 | | 2 - 21.0 - 47 | | 2 - 21.0 - 55 | |  |
| **Serum creatinine (mg/dL)** |  |  |  |  |  |  |  |  |  |  | Kruskal-Wallis : 0.002 |
| N | 69 | | 277 | | 361 | | 598 | | 1305 | |  |
| mean (std) | 1.4 (1.13) | | 1.3 (1.05) | | 1.5 (1.16) | | 1.8 (2.09) | | 1.6 (1.65) | |  |
| min - median - max | 0 - 1.0 - 6 | | 0 - 1.0 - 7 | | 0 - 1.1 - 8 | | 0 - 1.2 - 27 | | 0 - 1.1 - 27 | |  |
| **PaO²/FiO² (mmHg)** |  |  |  |  |  |  |  |  |  |  | Kruskal-Wallis : 0.307 |
| N | 67 | | 272 | | 338 | | 583 | | 1260 | |  |
| mean (std) | 215.1 (112.78) | | 224.5 (154.49) | | 217.5 (137.26) | | 205.1 (134.92) | | 213.1 (139.02) | |  |
| min - median - max | 48 - 190.0 - 564 | | 41 - 185.7 - 1356 | | 32 - 186.2 - 797 | | 34 - 182.5 - 1433 | | 32 - 185.0 - 1433 | |  |
| **Main cause of admission** |  |  |  |  |  |  |  |  |  |  | Chi-2 : 0.186 |
| Acute heart failure | 3 | (4.1%) | 17 | (5.9%) | 26 | (6.9%) | 47 | (7.5%) | 93 | (6.8%) |  |
| Sepsis/septic shock | 13 | (17.6%) | 42 | (14.6%) | 62 | (16.5%) | 123 | (19.7%) | 240 | (17.6%) |  |
| Acute kidney injury | 0 | (0.0%) | 2 | (0.7%) | 8 | (2.1%) | 18 | (2.9%) | 28 | (2.1%) |  |
| Post-surgery | 4 | (5.4%) | 11 | (3.8%) | 19 | (5.1%) | 22 | (3.5%) | 56 | (4.1%) |  |
| Acute respiratory distress syndrom (ARDS) | 31 | (41.9%) | 114 | (39.6%) | 152 | (40.5%) | 224 | (35.9%) | 521 | (38.3%) |  |
| Central nervous system injury | 22 | (29.7%) | 83 | (28.8%) | 90 | (24.0%) | 145 | (23.2%) | 340 | (25.0%) |  |
| Other | 1 | (1.4%) | 19 | (6.6%) | 18 | (4.8%) | 45 | (7.2%) | 83 | (6.1%) |  |
| Vasopressors at day 0 | 32 | (43.2%) | 153 | (53.1%) | 208 | (55.5%) | 359 | (57.5%) | 752 | (55.3%) | Chi-2 : 0.104 |
| RRT (#) at day 0 | 4 | (5.4%) | 7 | (2.4%) | 17 | (4.5%) | 59 | (9.5%) | 87 | (6.4%) | Chi-2 : <0.001 |

(*) SAPS : Simplified Acute Phyisiology Score
(~) SOFA : Sequential Organ Failure Assessment
(#) RRT : Renal-replacement therapy

# Table S2. Patients characteristics at admission according to ICU and intervention group (part 1 of 2)

|  | **ICU1** | | | | **ICU2** | | | | **ICU3** | | | | **ICU4** | | | | **ICU5** | | | | **ICU6** | | | |
| --- | --- | --- | --- | --- | --- | --- | --- | --- | --- | --- | --- | --- | --- | --- | --- | --- | --- | --- | --- | --- | --- | --- | --- | --- |
|  | Control | | Strategy | | Control | | Strategy | | Control | | Strategy | | Control | | Strategy | | Control | | Strategy | | Control | | Strategy | |
|  | N=61 | | N=57 | | N=104 | | N=58 | | N=35 | | N=25 | | N=46 | | N=48 | | N=44 | | N=59 | | N=71 | | N=71 | |
| **Age (years)** |  |  |  |  |  |  |  |  |  |  |  |  |  |  |  |  |  |  |  |  |  |  |  |  |
| N | 61 | | 57 | | 104 | | 58 | | 35 | | 25 | | 46 | | 48 | | 44 | | 59 | | 71 | | 71 | |
| mean (std) | 58.5 (16.53) | | 58.9 (13.64) | | 59.2 (15.79) | | 59.5 (15.26) | | 68.5 (11.74) | | 69.4 (13.74) | | 68.3 (13.39) | | 66.9 (13.71) | | 59.1 (12.03) | | 59.8 (13.92) | | 66.5 (15.75) | | 65.8 (13.53) | |
| min - median - max | 18 - 61.0 - 86 | | 19 - 62.0 - 84 | | 20 - 62.5 - 88 | | 27 - 62.0 - 84 | | 45 - 68.0 - 86 | | 29 - 70.0 - 93 | | 30 - 71.0 - 94 | | 31 - 70.5 - 86 | | 24 - 60.0 - 78 | | 22 - 61.0 - 83 | | 18 - 69.0 - 97 | | 28 - 67.0 - 88 | |
| **Male sex** | 38 | (62.3%) | 40 | (70.2%) | 71 | (68.3%) | 35 | (60.3%) | 24 | (68.6%) | 19 | (76.0%) | 29 | (63.0%) | 30 | (62.5%) | 21 | (47.7%) | 35 | (59.3%) | 47 | (66.2%) | 44 | (62.0%) |
| **Weight (kg)** |  |  |  |  |  |  |  |  |  |  |  |  |  |  |  |  |  |  |  |  |  |  |  |  |
| N | 61 | | 57 | | 104 | | 58 | | 35 | | 25 | | 46 | | 48 | | 44 | | 59 | | 71 | | 71 | |
| mean (std) | 78.3 (24.04) | | 78.8 (22.56) | | 78.7 (20.55) | | 81.1 (20.74) | | 79.4 (20.72) | | 81.7 (26.28) | | 74.5 (18.18) | | 80.9 (17.25) | | 80.0 (28.12) | | 73.3 (14.92) | | 75.5 (18.70) | | 80.5 (19.94) | |
| min - median - max | 38 - 75.0 - 148 | | 42 - 74.0 - 143 | | 45 - 77.0 - 156 | | 40 - 79.0 - 127 | | 37 - 80.0 - 128 | | 46 - 79.0 - 161 | | 41 - 71.5 - 137 | | 50 - 81.0 - 122 | | 42 - 75.0 - 204 | | 45 - 72.0 - 107 | | 34 - 71.0 - 135 | | 47 - 78.0 - 139 | |
| **Height (cm)** |  |  |  |  |  |  |  |  |  |  |  |  |  |  |  |  |  |  |  |  |  |  |  |  |
| N | 57 | | 52 | | 100 | | 57 | | 35 | | 24 | | 46 | | 48 | | 38 | | 46 | | 68 | | 71 | |
| mean (std) | 169.7 (10.26) | | 171.7 (8.37) | | 169.4 (9.41) | | 168.9 (10.47) | | 165.4 (11.01) | | 172.9 (11.53) | | 167.5 (10.18) | | 171.3 (7.50) | | 169.4 (8.98) | | 170.7 (11.07) | | 170.7 (10.43) | | 170.5 (9.47) | |
| min - median - max | 150 - 172.0 - 188 | | 155 - 172.5 - 190 | | 148 - 170.0 - 190 | | 145 - 170.0 - 195 | | 149 - 162.0 - 188 | | 149 - 176.0 - 189 | | 147 - 167.5 - 185 | | 155 - 171.0 - 187 | | 150 - 170.0 - 185 | | 150 - 170.5 - 196 | | 150 - 170.5 - 198 | | 145 - 172.0 - 190 | |
| **Body mass index (kg/m²)** |  |  |  |  |  |  |  |  |  |  |  |  |  |  |  |  |  |  |  |  |  |  |  |  |
| N | 57 | | 52 | | 100 | | 57 | | 35 | | 24 | | 46 | | 48 | | 38 | | 46 | | 68 | | 71 | |
| mean (std) | 27.7 (10.14) | | 26.5 (7.84) | | 27.5 (7.35) | | 28.5 (7.34) | | 29.1 (8.06) | | 27.4 (7.35) | | 26.5 (5.99) | | 27.6 (5.33) | | 28.0 (10.11) | | 25.1 (5.39) | | 25.7 (5.56) | | 27.7 (6.66) | |
| min - median - max | 15 - 25.7 - 62 | | 14 - 25.2 - 52 | | 16 - 25.8 - 59 | | 15 - 25.4 - 49 | | 17 - 27.3 - 50 | | 16 - 26.2 - 51 | | 16 - 25.4 - 47 | | 15 - 28.3 - 38 | | 16 - 26.2 - 71 | | 16 - 24.2 - 43 | | 15 - 25.1 - 42 | | 17 - 26.2 - 49 | |
| missing | 4 |  | 5 |  | 4 |  | 1 |  | 0 |  | 1 |  | 0 |  | 0 |  | 6 |  | 13 |  | 3 |  | 0 |  |
| Underweight | 10 | (16.4%) | 4 | (7.0%) | 4 | (3.8%) | 4 | (6.9%) | 1 | (2.9%) | 2 | (8.0%) | 1 | (2.2%) | 2 | (4.2%) | 3 | (6.8%) | 4 | (6.8%) | 7 | (9.9%) | 3 | (4.2%) |
| Normal | 14 | (23.0%) | 21 | (36.8%) | 36 | (34.6%) | 22 | (37.9%) | 10 | (28.6%) | 6 | (24.0%) | 20 | (43.5%) | 15 | (31.3%) | 15 | (34.1%) | 21 | (35.6%) | 27 | (38.0%) | 26 | (36.6%) |
| Overweight | 15 | (24.6%) | 15 | (26.3%) | 36 | (34.6%) | 7 | (12.1%) | 13 | (37.1%) | 11 | (44.0%) | 18 | (39.1%) | 12 | (25.0%) | 8 | (18.2%) | 13 | (22.0%) | 23 | (32.4%) | 19 | (26.8%) |
| Obese | 18 | (29.5%) | 12 | (21.1%) | 24 | (23.1%) | 24 | (41.4%) | 11 | (31.4%) | 5 | (20.0%) | 7 | (15.2%) | 19 | (39.6%) | 12 | (27.3%) | 8 | (13.6%) | 11 | (15.5%) | 23 | (32.4%) |
| **McCabe Score at admission** |  |  |  |  |  |  |  |  |  |  |  |  |  |  |  |  |  |  |  |  |  |  |  |  |
| Unknown | 10 |  | 6 |  | 9 |  | 2 |  | 1 |  | 4 |  | 4 |  | 7 |  | 3 |  | 1 |  | 4 |  | 3 |  |
| Non fatal | 37 | (60.7%) | 41 | (71.9%) | 76 | (73.1%) | 48 | (82.8%) | 22 | (62.9%) | 17 | (68.0%) | 21 | (45.7%) | 7 | (14.6%) | 39 | (88.6%) | 55 | (93.2%) | 30 | (42.3%) | 32 | (45.1%) |
| Ultimately fatal | 12 | (19.7%) | 7 | (12.3%) | 17 | (16.3%) | 8 | (13.8%) | 12 | (34.3%) | 3 | (12.0%) | 19 | (41.3%) | 26 | (54.2%) | 1 | (2.3%) | 3 | (5.1%) | 27 | (38.0%) | 28 | (39.4%) |
| Rapidly fatal | 2 | (3.3%) | 3 | (5.3%) | 2 | (1.9%) | 0 | (0.0%) | 0 | (0.0%) | 1 | (4.0%) | 2 | (4.3%) | 8 | (16.7%) | 1 | (2.3%) | 0 | (0.0%) | 10 | (14.1%) | 8 | (11.3%) |
| **Coexisting conditions** |  |  |  |  |  |  |  |  |  |  |  |  |  |  |  |  |  |  |  |  |  |  |  |  |
| Cirrhosis | 2 | (3.3%) | 5 | (8.8%) | 7 | (6.7%) | 2 | (3.4%) | 2 | (5.7%) | 1 | (4.0%) | 5 | (10.9%) | 4 | (8.3%) | 0 | (0.0%) | 1 | (1.7%) | 6 | (8.5%) | 6 | (8.5%) |
| Cancer | 6 | (9.8%) | 11 | (19.3%) | 8 | (7.7%) | 4 | (6.9%) | 3 | (8.6%) | 3 | (12.0%) | 8 | (17.4%) | 5 | (10.4%) | 2 | (4.5%) | 6 | (10.2%) | 23 | (32.4%) | 25 | (35.2%) |
| Immunodeficiency | 10 | (16.4%) | 11 | (19.3%) | 7 | (6.7%) | 4 | (6.9%) | 5 | (14.3%) | 2 | (8.0%) | 6 | (13.0%) | 4 | (8.3%) | 2 | (4.5%) | 8 | (13.6%) | 14 | (19.7%) | 16 | (22.5%) |
| Heart failure | 11 | (18.0%) | 9 | (15.8%) | 4 | (3.8%) | 3 | (5.2%) | 8 | (22.9%) | 2 | (8.0%) | 4 | (8.7%) | 2 | (4.2%) | 1 | (2.3%) | 3 | (5.1%) | 2 | (2.8%) | 4 | (5.6%) |
| Diabetes mellitus | 17 | (27.9%) | 9 | (15.8%) | 22 | (21.2%) | 8 | (13.8%) | 10 | (28.6%) | 8 | (32.0%) | 11 | (23.9%) | 16 | (33.3%) | 6 | (13.6%) | 10 | (16.9%) | 18 | (25.4%) | 18 | (25.4%) |
| Chronic respiratory failure | 11 | (18.0%) | 11 | (19.3%) | 14 | (13.5%) | 7 | (12.1%) | 19 | (54.3%) | 10 | (40.0%) | 11 | (23.9%) | 6 | (12.5%) | 4 | (9.1%) | 7 | (11.9%) | 8 | (11.3%) | 13 | (18.3%) |
| Chronic kidney disease under RRT (#) | 3 | (4.9%) | 0 | (0.0%) | 0 | (0.0%) | 0 | (0.0%) | 0 | (0.0%) | 0 | (0.0%) | 1 | (2.2%) | 0 | (0.0%) | 0 | (0.0%) | 0 | (0.0%) | 5 | (7.0%) | 1 | (1.4%) |
| **SAPS II at admission (*)** |  |  |  |  |  |  |  |  |  |  |  |  |  |  |  |  |  |  |  |  |  |  |  |  |
| N | 61 | | 57 | | 104 | | 53 | | 35 | | 25 | | 46 | | 48 | | 44 | | 55 | | 71 | | 71 | |
| mean (std) | 56.3 (17.66) | | 53.2 (18.70) | | 56.1 (18.75) | | 56.4 (18.09) | | 58.1 (17.66) | | 54.6 (23.80) | | 57.5 (18.13) | | 56.2 (19.41) | | 48.1 (11.81) | | 53.0 (11.12) | | 61.7 (15.57) | | 59.0 (18.47) | |
| min - median - max | 25 - 55.0 - 96 | | 21 - 52.0 - 103 | | 15 - 56.5 - 115 | | 18 - 59.0 - 88 | | 33 - 55.0 - 98 | | 14 - 48.0 - 98 | | 24 - 55.5 - 99 | | 21 - 56.0 - 93 | | 21 - 47.0 - 77 | | 24 - 55.0 - 84 | | 28 - 63.0 - 113 | | 31 - 58.0 - 131 | |
| **SOFA (~)** |  |  |  |  |  |  |  |  |  |  |  |  |  |  |  |  |  |  |  |  |  |  |  |  |
| N | 61 | | 57 | | 104 | | 58 | | 35 | | 25 | | 46 | | 48 | | 44 | | 59 | | 71 | | 71 | |
| mean (std) | 8.8 (3.57) | | 9.3 (3.94) | | 9.1 (4.18) | | 8.8 (2.84) | | 10.1 (3.73) | | 9.3 (4.70) | | 9.5 (4.41) | | 8.6 (3.34) | | 8.8 (3.12) | | 8.3 (2.89) | | 9.1 (3.91) | | 9.9 (3.60) | |
| min - median - max | 2 - 8.0 - 16 | | 1 - 9.0 - 19 | | 0 - 9.0 - 20 | | 1 - 9.0 - 13 | | 3 - 10.0 - 16 | | 0 - 8.0 - 18 | | 0 - 10.0 - 18 | | 2 - 8.0 - 16 | | 3 - 9.0 - 17 | | 3 - 8.0 - 15 | | 1 - 9.0 - 16 | | 2 - 10.0 - 18 | |
| **Serum sodium (mmol/L)** |  |  |  |  |  |  |  |  |  |  |  |  |  |  |  |  |  |  |  |  |  |  |  |  |
| N | 60 | | 56 | | 101 | | 58 | | 33 | | 23 | | 44 | | 44 | | 44 | | 59 | | 70 | | 66 | |
| mean (std) | 136.7 (6.65) | | 136.2 (7.58) | | 136.6 (6.30) | | 137.4 (5.27) | | 139.4 (4.86) | | 140.3 (4.14) | | 140.4 (7.30) | | 139.0 (7.59) | | 136.4 (5.56) | | 139.0 (6.89) | | 141.2 (6.86) | | 140.7 (6.36) | |
| min - median - max | 120 - 137.0 - 160 | | 116 - 136.5 - 153 | | 116 - 137.0 - 156 | | 123 - 137.0 - 152 | | 131 - 138.0 - 150 | | 135 - 139.0 - 150 | | 129 - 139.0 - 162 | | 125 - 139.0 - 172 | | 121 - 136.0 - 149 | | 126 - 138.0 - 157 | | 120 - 140.0 - 163 | | 125 - 140.0 - 161 | |
| **Serum potassium (mmol/L)** |  |  |  |  |  |  |  |  |  |  |  |  |  |  |  |  |  |  |  |  |  |  |  |  |
| N | 60 | | 56 | | 102 | | 58 | | 33 | | 23 | | 42 | | 44 | | 44 | | 59 | | 70 | | 67 | |
| mean (std) | 4.1 (0.85) | | 4.1 (1.05) | | 4.1 (0.79) | | 4.0 (0.79) | | 4.3 (0.76) | | 4.6 (0.85) | | 4.1 (0.82) | | 4.0 (0.88) | | 3.9 (0.94) | | 3.8 (0.77) | | 4.1 (1.16) | | 3.9 (1.01) | |
| min - median - max | 2 - 4.0 - 6 | | 2 - 3.8 - 9 | | 3 - 4.0 - 6 | | 3 - 3.9 - 7 | | 3 - 4.2 - 6 | | 3 - 4.6 - 7 | | 3 - 4.1 - 7 | | 3 - 4.0 - 7 | | 3 - 3.7 - 7 | | 2 - 3.7 - 6 | | 2 - 3.9 - 8 | | 1 - 3.9 - 6 | |
| **Serum bicarbonate (mmol/L)** |  |  |  |  |  |  |  |  |  |  |  |  |  |  |  |  |  |  |  |  |  |  |  |  |
| N | 57 | | 55 | | 98 | | 52 | | 33 | | 20 | | 44 | | 44 | | 38 | | 58 | | 67 | | 66 | |
| mean (std) | 22.5 (7.96) | | 21.5 (6.67) | | 21.1 (5.25) | | 23.1 (6.71) | | 23.8 (5.49) | | 24.0 (5.39) | | 21.2 (7.16) | | 22.9 (5.55) | | 22.8 (6.14) | | 20.9 (4.46) | | 19.9 (6.29) | | 21.3 (6.72) | |
| min - median - max | 3 - 21.9 - 55 | | 6 - 20.6 - 35 | | 7 - 21.0 - 35 | | 12 - 22.6 - 42 | | 14 - 23.3 - 36 | | 11 - 25.5 - 32 | | 6 - 19.8 - 44 | | 8 - 23.0 - 37 | | 7 - 23.6 - 40 | | 7 - 21.0 - 33 | | 3 - 19.4 - 37 | | 5 - 21.1 - 41 | |
| **Serum creatinine (mg/dL)** |  |  |  |  |  |  |  |  |  |  |  |  |  |  |  |  |  |  |  |  |  |  |  |  |
| N | 58 | | 51 | | 96 | | 58 | | 33 | | 23 | | 44 | | 44 | | 44 | | 57 | | 69 | | 66 | |
| mean (std) | 1.5 (1.71) | | 2.0 (2.56) | | 1.7 (1.22) | | 1.3 (1.07) | | 1.5 (1.26) | | 1.3 (0.87) | | 1.7 (1.05) | | 1.4 (1.12) | | 1.0 (1.05) | | 1.0 (0.93) | | 1.6 (1.63) | | 1.7 (1.44) | |
| min - median - max | 0 - 1.0 - 12 | | 0 - 1.1 - 18 | | 1 - 1.3 - 7 | | 0 - 1.0 - 7 | | 0 - 1.0 - 6 | | 0 - 0.8 - 4 | | 0 - 1.3 - 4 | | 0 - 1.0 - 5 | | 0 - 0.7 - 7 | | 0 - 0.7 - 6 | | 0 - 1.0 - 8 | | 0 - 1.2 - 8 | |
| **PaO²/FiO² (mmHg)** |  |  |  |  |  |  |  |  |  |  |  |  |  |  |  |  |  |  |  |  |  |  |  |  |
| N | 58 | | 53 | | 96 | | 49 | | 33 | | 20 | | 44 | | 46 | | 44 | | 59 | | 68 | | 65 | |
| mean (std) | 188.1 (99.39) | | 176.6 (103.49) | | 248.7 (199.42) | | 211.1 (114.85) | | 158.0 (78.02) | | 190.7 (102.58) | | 222.3 (144.16) | | 207.3 (124.50) | | 229.6 (124.15) | | 241.7 (113.55) | | 248.5 (107.89) | | 214.6 (114.37) | |
| min - median - max | 42 - 172.5 - 443 | | 48 - 151.7 - 481 | | 45 - 200.0 - 1356 | | 37 - 190.0 - 620 | | 57 - 145.0 - 366 | | 74 - 156.3 - 402 | | 56 - 206.0 - 585 | | 38 - 197.9 - 560 | | 52 - 197.5 - 492 | | 58 - 242.0 - 475 | | 49 - 238.3 - 629 | | 34 - 200.0 - 624 | |
| **Main cause of admission** |  |  |  |  |  |  |  |  |  |  |  |  |  |  |  |  |  |  |  |  |  |  |  |  |
| Acute heart failure | 2 | (3.3%) | 2 | (3.5%) | 19 | (18.3%) | 5 | (8.6%) | 1 | (2.9%) | 1 | (4.0%) | 2 | (4.3%) | 0 | (0.0%) | 0 | (0.0%) | 0 | (0.0%) | 1 | (1.4%) | 5 | (7.0%) |
| Sepsis/septic shock | 16 | (26.2%) | 8 | (14.0%) | 12 | (11.5%) | 8 | (13.8%) | 6 | (17.1%) | 3 | (12.0%) | 11 | (23.9%) | 7 | (14.6%) | 4 | (9.1%) | 4 | (6.8%) | 25 | (35.2%) | 18 | (25.4%) |
| Acute kidney injury | 0 | (0.0%) | 0 | (0.0%) | 4 | (3.8%) | 2 | (3.4%) | 1 | (2.9%) | 0 | (0.0%) | 0 | (0.0%) | 1 | (2.1%) | 0 | (0.0%) | 0 | (0.0%) | 1 | (1.4%) | 1 | (1.4%) |
| Post-surgery | 2 | (3.3%) | 2 | (3.5%) | 6 | (5.8%) | 4 | (6.9%) | 1 | (2.9%) | 0 | (0.0%) | 0 | (0.0%) | 0 | (0.0%) | 0 | (0.0%) | 7 | (11.9%) | 1 | (1.4%) | 4 | (5.6%) |
| Acute respiratory distress syndrom (ARDS) | 17 | (27.9%) | 19 | (33.3%) | 44 | (42.3%) | 21 | (36.2%) | 22 | (62.9%) | 14 | (56.0%) | 28 | (60.9%) | 22 | (45.8%) | 4 | (9.1%) | 2 | (3.4%) | 21 | (29.6%) | 24 | (33.8%) |
| Central nervous system injury | 24 | (39.3%) | 23 | (40.4%) | 11 | (10.6%) | 16 | (27.6%) | 4 | (11.4%) | 7 | (28.0%) | 4 | (8.7%) | 14 | (29.2%) | 32 | (72.7%) | 43 | (72.9%) | 18 | (25.4%) | 12 | (16.9%) |
| Other | 0 | (0.0%) | 3 | (5.3%) | 8 | (7.7%) | 2 | (3.4%) | 0 | (0.0%) | 0 | (0.0%) | 1 | (2.2%) | 4 | (8.3%) | 4 | (9.1%) | 3 | (5.1%) | 4 | (5.6%) | 7 | (9.9%) |
| **Vasopressors at Day0** | 26 | (42.6%) | 28 | (49.1%) | 54 | (51.9%) | 27 | (46.6%) | 21 | (60.0%) | 13 | (52.0%) | 26 | (56.5%) | 25 | (52.1%) | 26 | (59.1%) | 32 | (54.2%) | 35 | (49.3%) | 47 | (66.2%) |
| **RRT (#) at Day0** | 4 | (6.6%) | 6 | (10.5%) | 10 | (9.6%) | 1 | (1.7%) | 1 | (2.9%) | 3 | (12.0%) | 3 | (6.5%) | 0 | (0.0%) | 1 | (2.3%) | 2 | (3.4%) | 2 | (2.8%) | 7 | (9.9%) |

(*) SAPS : Simplified Acute Phyisiology Score
(~) SOFA : Sequential Organ Failure Assessment
(#) RRT : Renal-replacement therapy

# Table S2. Patients characteristics at admission according to ICU and intervention group (part 2 of 2)

**Table S1.2 Description of patients' characteristics at admission**

|  | **ICU7** | | | | **ICU8** | | | | **ICU9** | | | | **ICU10** | | | | **ICU11** | | | | **ICU12** | | | |
| --- | --- | --- | --- | --- | --- | --- | --- | --- | --- | --- | --- | --- | --- | --- | --- | --- | --- | --- | --- | --- | --- | --- | --- | --- |
|  | Control | | Strategy | | Control | | Strategy | | Control | | Strategy | | Control | | Strategy | | Control | | Strategy | | Control | | Strategy | |
|  | N=35 | | N=29 | | N=91 | | N=86 | | N=86 | | N=47 | | N=52 | | N=49 | | N=44 | | N=49 | | N=49 | | N=65 | |
| **Age (years)** |  |  |  |  |  |  |  |  |  |  |  |  |  |  |  |  |  |  |  |  |  |  |  |  |
| N | 35 | | 29 | | 91 | | 86 | | 86 | | 47 | | 52 | | 49 | | 44 | | 49 | | 49 | | 65 | |
| mean (std) | 69.4 (17.16) | | 71.6 (10.26) | | 67.2 (11.66) | | 64.4 (13.61) | | 66.6 (13.85) | | 63.7 (15.17) | | 66.2 (12.77) | | 68.9 (11.51) | | 63.2 (17.55) | | 62.9 (16.55) | | 65.4 (13.75) | | 66.6 (13.82) | |
| min - median - max | 24 - 73.0 - 92 | | 55 - 71.0 - 91 | | 32 - 69.0 - 89 | | 29 - 66.0 - 92 | | 23 - 70.0 - 86 | | 31 - 65.0 - 86 | | 33 - 66.5 - 87 | | 38 - 70.0 - 88 | | 19 - 66.0 - 89 | | 19 - 66.0 - 84 | | 32 - 68.0 - 84 | | 19 - 68.0 - 88 | |
| **Male sex** | 27 | (77.1%) | 15 | (51.7%) | 66 | (72.5%) | 60 | (69.8%) | 54 | (62.8%) | 30 | (63.8%) | 31 | (59.6%) | 32 | (65.3%) | 28 | (63.6%) | 31 | (63.3%) | 34 | (69.4%) | 38 | (58.5%) |
| **Weight (kg)** |  |  |  |  |  |  |  |  |  |  |  |  |  |  |  |  |  |  |  |  |  |  |  |  |
| N | 35 | | 29 | | 91 | | 86 | | 86 | | 47 | | 52 | | 49 | | 44 | | 49 | | 49 | | 65 | |
| mean (std) | 72.9 (15.13) | | 66.1 (10.39) | | 82.7 (26.41) | | 80.6 (21.28) | | 76.0 (19.64) | | 76.1 (23.41) | | 87.9 (25.91) | | 82.9 (17.80) | | 78.2 (24.27) | | 84.5 (24.67) | | 81.1 (24.62) | | 75.4 (22.42) | |
| min - median - max | 40 - 77.0 - 97 | | 47 - 65.0 - 92 | | 43 - 78.0 - 232 | | 39 - 81.0 - 164 | | 35 - 76.0 - 138 | | 49 - 70.0 - 155 | | 52 - 82.5 - 174 | | 54 - 84.0 - 135 | | 41 - 71.0 - 179 | | 42 - 80.0 - 172 | | 29 - 80.0 - 148 | | 39 - 72.0 - 142 | |
| **Height (cm)** |  |  |  |  |  |  |  |  |  |  |  |  |  |  |  |  |  |  |  |  |  |  |  |  |
| N | 34 | | 29 | | 90 | | 86 | | 84 | | 46 | | 48 | | 48 | | 43 | | 48 | | 46 | | 64 | |
| mean (std) | 169.5 (9.61) | | 164.5 (9.31) | | 170.9 (8.97) | | 169.6 (8.10) | | 169.5 (10.20) | | 166.9 (9.64) | | 169.5 (11.04) | | 167.9 (10.14) | | 168.1 (7.97) | | 170.6 (9.66) | | 169.5 (7.99) | | 167.7 (9.54) | |
| min - median - max | 151 - 169.5 - 190 | | 142 - 165.0 - 180 | | 148 - 172.0 - 195 | | 151 - 169.5 - 192 | | 143 - 170.0 - 193 | | 144 - 168.0 - 181 | | 135 - 170.0 - 192 | | 143 - 170.0 - 186 | | 150 - 170.0 - 188 | | 150 - 170.0 - 200 | | 145 - 170.0 - 185 | | 143 - 167.0 - 185 | |
| **Body mass index (kg/m²)** |  |  |  |  |  |  |  |  |  |  |  |  |  |  |  |  |  |  |  |  |  |  |  |  |
| N | 34 | | 29 | | 90 | | 86 | | 84 | | 46 | | 48 | | 48 | | 43 | | 48 | | 46 | | 64 | |
| mean (std) | 25.2 (4.02) | | 24.6 (4.24) | | 28.3 (8.33) | | 28.0 (7.24) | | 26.2 (6.05) | | 27.7 (9.87) | | 30.3 (8.71) | | 29.6 (7.17) | | 27.4 (7.70) | | 29.6 (9.35) | | 27.9 (8.44) | | 26.7 (7.46) | |
| min - median - max | 17 - 24.6 - 34 | | 17 - 24.5 - 37 | | 17 - 26.2 - 76 | | 15 - 26.8 - 57 | | 14 - 25.9 - 51 | | 16 - 26.0 - 60 | | 17 - 28.8 - 64 | | 20 - 28.0 - 51 | | 18 - 25.7 - 58 | | 15 - 28.0 - 66 | | 14 - 25.7 - 54 | | 14 - 24.8 - 49 | |
| missing | 1 |  | 0 |  | 1 |  | 0 |  | 2 |  | 1 |  | 4 |  | 1 |  | 1 |  | 1 |  | 3 |  | 1 |  |
| Underweight | 3 | (8.6%) | 1 | (3.4%) | 4 | (4.4%) | 5 | (5.8%) | 7 | (8.1%) | 5 | (10.6%) | 2 | (3.8%) | 0 | (0.0%) | 1 | (2.3%) | 2 | (4.1%) | 3 | (6.1%) | 7 | (10.8%) |
| Normal | 15 | (42.9%) | 17 | (58.6%) | 35 | (38.5%) | 27 | (31.4%) | 26 | (30.2%) | 14 | (29.8%) | 12 | (23.1%) | 14 | (28.6%) | 19 | (43.2%) | 13 | (26.5%) | 19 | (38.8%) | 26 | (40.0%) |
| Overweight | 13 | (37.1%) | 9 | (31.0%) | 20 | (22.0%) | 25 | (29.1%) | 30 | (34.9%) | 17 | (36.2%) | 17 | (32.7%) | 16 | (32.7%) | 11 | (25.0%) | 18 | (36.7%) | 8 | (16.3%) | 8 | (12.3%) |
| Obese | 3 | (8.6%) | 2 | (6.9%) | 31 | (34.1%) | 29 | (33.7%) | 21 | (24.4%) | 10 | (21.3%) | 17 | (32.7%) | 18 | (36.7%) | 12 | (27.3%) | 15 | (30.6%) | 16 | (32.7%) | 23 | (35.4%) |
| **McCabe Score at admission** |  |  |  |  |  |  |  |  |  |  |  |  |  |  |  |  |  |  |  |  |  |  |  |  |
| missing | 0 |  | 0 |  | 0 |  | 0 |  | 0 |  | 0 |  | 0 |  | 0 |  | 0 |  | 1 |  | 0 |  | 0 |  |
| Unknown | 1 |  | 0 |  | 22 |  | 8 |  | 0 |  | 4 |  | 1 |  | 0 |  | 1 |  | 0 |  | 0 |  | 0 |  |
| Non fatal | 27 | (77.1%) | 23 | (79.3%) | 14 | (15.4%) | 56 | (65.1%) | 53 | (61.6%) | 34 | (72.3%) | 38 | (73.1%) | 35 | (71.4%) | 29 | (65.9%) | 27 | (55.1%) | 33 | (67.3%) | 53 | (81.5%) |
| Ultimately fatal | 5 | (14.3%) | 6 | (20.7%) | 50 | (54.9%) | 17 | (19.8%) | 23 | (26.7%) | 8 | (17.0%) | 11 | (21.2%) | 10 | (20.4%) | 10 | (22.7%) | 17 | (34.7%) | 16 | (32.7%) | 10 | (15.4%) |
| Rapidly fatal | 2 | (5.7%) | 0 | (0.0%) | 5 | (5.5%) | 5 | (5.8%) | 10 | (11.6%) | 1 | (2.1%) | 2 | (3.8%) | 4 | (8.2%) | 4 | (9.1%) | 4 | (8.2%) | 0 | (0.0%) | 2 | (3.1%) |
| **Coexisting conditions** |  |  |  |  |  |  |  |  |  |  |  |  |  |  |  |  |  |  |  |  |  |  |  |  |
| Cirrhosis | 1 | (2.9%) | 1 | (3.4%) | 10 | (11.0%) | 6 | (7.0%) | 6 | (7.0%) | 7 | (14.9%) | 2 | (3.8%) | 3 | (6.1%) | 3 | (6.8%) | 3 | (6.1%) | 4 | (8.2%) | 7 | (10.8%) |
| Cancer | 7 | (20.0%) | 6 | (20.7%) | 16 | (17.6%) | 18 | (20.9%) | 15 | (17.4%) | 5 | (10.6%) | 3 | (5.8%) | 5 | (10.2%) | 6 | (13.6%) | 6 | (12.2%) | 6 | (12.2%) | 5 | (7.7%) |
| Immunodeficiency | 3 | (8.6%) | 3 | (10.3%) | 13 | (14.3%) | 15 | (17.4%) | 19 | (22.1%) | 5 | (10.6%) | 5 | (9.6%) | 4 | (8.2%) | 3 | (6.8%) | 3 | (6.1%) | 5 | (10.2%) | 3 | (4.6%) |
| Heart failure | 0 | (0.0%) | 0 | (0.0%) | 15 | (16.5%) | 14 | (16.3%) | 5 | (5.8%) | 2 | (4.3%) | 1 | (1.9%) | 1 | (2.0%) | 10 | (22.7%) | 7 | (14.3%) | 4 | (8.2%) | 11 | (16.9%) |
| Diabetes mellitus | 10 | (28.6%) | 6 | (20.7%) | 31 | (34.1%) | 27 | (31.4%) | 28 | (32.6%) | 13 | (27.7%) | 18 | (34.6%) | 14 | (28.6%) | 11 | (25.0%) | 15 | (30.6%) | 9 | (18.4%) | 20 | (30.8%) |
| Chronic respiratory failure | 2 | (5.7%) | 4 | (13.8%) | 20 | (22.0%) | 22 | (25.6%) | 18 | (20.9%) | 4 | (8.5%) | 17 | (32.7%) | 23 | (46.9%) | 7 | (15.9%) | 13 | (26.5%) | 16 | (32.7%) | 24 | (36.9%) |
| Chronic kidney disease under RRT (#) | 1 | (2.9%) | 4 | (13.8%) | 3 | (3.3%) | 3 | (3.5%) | 1 | (1.2%) | 1 | (2.1%) | 1 | (1.9%) | 1 | (2.0%) | 1 | (2.3%) | 1 | (2.0%) | 0 | (0.0%) | 0 | (0.0%) |
| **SAPS II at admission (*)** |  |  |  |  |  |  |  |  |  |  |  |  |  |  |  |  |  |  |  |  |  |  |  |  |
| N | 35 | | 29 | | 90 | | 86 | | 86 | | 47 | | 52 | | 49 | | 44 | | 49 | | 49 | | 64 | |
| mean (std) | 65.4 (18.05) | | 65.5 (19.59) | | 64.3 (18.28) | | 61.2 (18.17) | | 59.0 (17.14) | | 58.4 (15.42) | | 53.0 (18.45) | | 64.2 (19.43) | | 62.2 (20.19) | | 62.1 (18.54) | | 65.4 (15.51) | | 58.1 (17.34) | |
| min - median - max | 17 - 62.0 - 101 | | 33 - 68.0 - 102 | | 22 - 65.0 - 110 | | 26 - 60.0 - 99 | | 30 - 59.5 - 103 | | 37 - 55.0 - 93 | | 13 - 50.5 - 105 | | 26 - 64.0 - 99 | | 12 - 58.5 - 106 | | 18 - 65.0 - 109 | | 35 - 65.0 - 109 | | 23 - 57.5 - 100 | |
| **SOFA (~)** |  |  |  |  |  |  |  |  |  |  |  |  |  |  |  |  |  |  |  |  |  |  |  |  |
| N | 35 | | 29 | | 91 | | 86 | | 86 | | 47 | | 52 | | 49 | | 44 | | 49 | | 49 | | 65 | |
| mean (std) | 9.2 (3.75) | | 8.6 (3.79) | | 8.7 (4.24) | | 7.5 (3.13) | | 9.3 (4.25) | | 9.4 (3.77) | | 8.0 (3.72) | | 8.4 (3.44) | | 10.4 (3.62) | | 9.5 (4.15) | | 8.5 (3.40) | | 8.0 (3.68) | |
| min - median - max | 0 - 9.0 - 15 | | 0 - 9.0 - 16 | | 1 - 9.0 - 20 | | 1 - 8.0 - 15 | | 0 - 10.0 - 19 | | 1 - 9.0 - 17 | | 2 - 9.0 - 17 | | 2 - 9.0 - 16 | | 3 - 11.0 - 18 | | 2 - 10.0 - 18 | | 0 - 8.0 - 16 | | 2 - 8.0 - 16 | |
| **Serum sodium (mmol/L)** |  |  |  |  |  |  |  |  |  |  |  |  |  |  |  |  |  |  |  |  |  |  |  |  |
| N | 32 | | 27 | | 90 | | 86 | | 80 | | 46 | | 52 | | 49 | | 44 | | 49 | | 48 | | 64 | |
| mean (std) | 137.4 (7.33) | | 135.4 (6.59) | | 136.4 (7.32) | | 136.8 (5.77) | | 138.3 (5.59) | | 139.2 (7.01) | | 135.6 (6.05) | | 137.1 (7.63) | | 138.1 (6.68) | | 135.6 (7.89) | | 138.6 (6.48) | | 136.9 (5.31) | |
| min - median - max | 118 - 138.5 - 152 | | 114 - 135.0 - 150 | | 123 - 135.5 - 174 | | 120 - 137.0 - 155 | | 113 - 138.0 - 155 | | 124 - 139.0 - 156 | | 120 - 136.0 - 150 | | 120 - 136.0 - 154 | | 106 - 138.5 - 152 | | 114 - 137.0 - 161 | | 125 - 138.0 - 159 | | 119 - 137.5 - 146 | |
| **Serum potassium (mmol/L)** |  |  |  |  |  |  |  |  |  |  |  |  |  |  |  |  |  |  |  |  |  |  |  |  |
| N | 31 | | 27 | | 91 | | 84 | | 79 | | 46 | | 52 | | 49 | | 44 | | 48 | | 47 | | 64 | |
| mean (std) | 4.2 (0.76) | | 4.2 (1.05) | | 4.3 (0.89) | | 4.1 (0.84) | | 4.3 (0.80) | | 4.1 (0.98) | | 4.4 (1.09) | | 4.3 (1.24) | | 4.2 (1.15) | | 4.1 (0.96) | | 4.4 (1.01) | | 4.3 (0.91) | |
| min - median - max | 3 - 4.2 - 5 | | 2 - 4.1 - 7 | | 3 - 4.1 - 7 | | 3 - 4.0 - 7 | | 3 - 4.2 - 7 | | 2 - 4.0 - 7 | | 2 - 4.2 - 7 | | 3 - 4.0 - 9 | | 3 - 3.9 - 7 | | 2 - 4.1 - 7 | | 3 - 4.2 - 8 | | 2 - 4.3 - 6 | |
| **Serum bicarbonate (mmol/L)** |  |  |  |  |  |  |  |  |  |  |  |  |  |  |  |  |  |  |  |  |  |  |  |  |
| N | 29 | | 25 | | 86 | | 82 | | 82 | | 46 | | 51 | | 49 | | 44 | | 49 | | 42 | | 60 | |
| mean (std) | 19.6 (7.00) | | 20.2 (6.27) | | 21.2 (7.91) | | 22.4 (7.25) | | 20.5 (6.31) | | 20.2 (5.74) | | 22.1 (7.05) | | 23.7 (9.03) | | 17.5 (7.95) | | 22.0 (9.09) | | 23.1 (9.71) | | 24.7 (8.40) | |
| min - median - max | 9 - 19.0 - 36 | | 8 - 20.0 - 41 | | 4 - 20.9 - 46 | | 5 - 21.0 - 41 | | 2 - 21.0 - 36 | | 6 - 20.0 - 35 | | 4 - 22.0 - 35 | | 4 - 23.0 - 47 | | 4 - 17.7 - 41 | | 4 - 21.0 - 46 | | 3 - 21.7 - 44 | | 2 - 24.8 - 42 | |
| **Serum creatinine (mg/dL)** |  |  |  |  |  |  |  |  |  |  |  |  |  |  |  |  |  |  |  |  |  |  |  |  |
| N | 32 | | 26 | | 91 | | 86 | | 81 | | 42 | | 52 | | 48 | | 44 | | 49 | | 47 | | 64 | |
| mean (std) | 1.7 (1.46) | | 1.6 (1.24) | | 1.7 (1.24) | | 1.4 (1.04) | | 2.0 (3.29) | | 1.6 (1.61) | | 1.7 (1.56) | | 1.8 (1.43) | | 1.8 (1.75) | | 2.3 (2.62) | | 1.7 (1.35) | | 1.4 (1.10) | |
| min - median - max | 0 - 1.4 - 8 | | 0 - 1.3 - 7 | | 0 - 1.2 - 5 | | 0 - 1.1 - 5 | | 0 - 1.1 - 27 | | 0 - 1.0 - 8 | | 0 - 1.1 - 8 | | 1 - 1.4 - 7 | | 0 - 1.3 - 11 | | 0 - 1.5 - 17 | | 0 - 1.4 - 8 | | 0 - 1.0 - 5 | |
| **PaO²/FiO² (mmHg)** |  |  |  |  |  |  |  |  |  |  |  |  |  |  |  |  |  |  |  |  |  |  |  |  |
| N | 25 | | 21 | | 83 | | 81 | | 76 | | 41 | | 48 | | 49 | | 44 | | 48 | | 46 | | 63 | |
| mean (std) | 266.9 (180.07) | | 224.3 (145.47) | | 227.1 (150.93) | | 228.9 (184.94) | | 209.8 (183.61) | | 205.1 (130.93) | | 168.7 (83.55) | | 174.4 (97.02) | | 218.8 (125.88) | | 194.8 (121.47) | | 172.4 (116.10) | | 219.8 (135.33) | |
| min - median - max | 53 - 248.3 - 797 | | 47 - 186.7 - 543 | | 45 - 177.1 - 878 | | 41 - 194.0 - 1433 | | 42 - 149.0 - 1104 | | 44 - 190.0 - 727 | | 32 - 165.8 - 430 | | 41 - 136.0 - 485 | | 53 - 185.5 - 573 | | 43 - 167.5 - 505 | | 41 - 135.7 - 518 | | 37 - 176.0 - 548 | |
| **Main cause of admission** |  |  |  |  |  |  |  |  |  |  |  |  |  |  |  |  |  |  |  |  |  |  |  |  |
| Acute heart failure | 4 | (11.4%) | 1 | (3.4%) | 15 | (16.5%) | 8 | (9.3%) | 4 | (4.7%) | 2 | (4.3%) | 2 | (3.8%) | 0 | (0.0%) | 5 | (11.4%) | 4 | (8.2%) | 5 | (10.2%) | 5 | (7.7%) |
| Sepsis/septic shock | 4 | (11.4%) | 4 | (13.8%) | 18 | (19.8%) | 18 | (20.9%) | 12 | (14.0%) | 8 | (17.0%) | 7 | (13.5%) | 7 | (14.3%) | 10 | (22.7%) | 7 | (14.3%) | 8 | (16.3%) | 15 | (23.1%) |
| Acute kidney injury | 2 | (5.7%) | 0 | (0.0%) | 1 | (1.1%) | 4 | (4.7%) | 3 | (3.5%) | 1 | (2.1%) | 0 | (0.0%) | 3 | (6.1%) | 3 | (6.8%) | 0 | (0.0%) | 0 | (0.0%) | 1 | (1.5%) |
| Post-surgery | 4 | (11.4%) | 3 | (10.3%) | 0 | (0.0%) | 0 | (0.0%) | 1 | (1.2%) | 1 | (2.1%) | 4 | (7.7%) | 6 | (12.2%) | 2 | (4.5%) | 2 | (4.1%) | 2 | (4.1%) | 4 | (6.2%) |
| Acute respiratory distress syndrom (ARDS) | 8 | (22.9%) | 15 | (51.7%) | 30 | (33.0%) | 34 | (39.5%) | 43 | (50.0%) | 19 | (40.4%) | 25 | (48.1%) | 25 | (51.0%) | 12 | (27.3%) | 14 | (28.6%) | 29 | (59.2%) | 29 | (44.6%) |
| Central nervous system injury | 11 | (31.4%) | 5 | (17.2%) | 18 | (19.8%) | 19 | (22.1%) | 16 | (18.6%) | 10 | (21.3%) | 11 | (21.2%) | 7 | (14.3%) | 9 | (20.5%) | 17 | (34.7%) | 3 | (6.1%) | 6 | (9.2%) |
| Other | 2 | (5.7%) | 1 | (3.4%) | 9 | (9.9%) | 3 | (3.5%) | 7 | (8.1%) | 6 | (12.8%) | 3 | (5.8%) | 1 | (2.0%) | 3 | (6.8%) | 5 | (10.2%) | 2 | (4.1%) | 5 | (7.7%) |
| **Vasopressors at day 0** | 19 | (54.3%) | 13 | (44.8%) | 51 | (56.0%) | 51 | (59.3%) | 62 | (72.1%) | 33 | (70.2%) | 18 | (34.6%) | 25 | (51.0%) | 27 | (61.4%) | 28 | (57.1%) | 30 | (61.2%) | 35 | (53.8%) |
| **RRT (#) at day 0** | 2 | (5.7%) | 1 | (3.4%) | 12 | (13.2%) | 5 | (5.8%) | 5 | (5.8%) | 3 | (6.4%) | 4 | (7.7%) | 3 | (6.1%) | 4 | (9.1%) | 4 | (8.2%) | 3 | (6.1%) | 1 | (1.5%) |
| (*) SAPS : Simplified Acute Phyisiology Score (~) SOFA : Sequential Organ Failure Assessment (#) RRT : Renal-replacement therapy | | | | | | | | | | | | | | | | | | | | | | | | |

# Table S3. Description of missing values

| **Characteristic** | **Number of missing data** | **% of missing data** |
| --- | --- | --- |
| Age (years) | 0 | 0 |
| Gender | 0 | 0 |
| Weight (kg) | 0 | 0 |
| Height (cm) | 53 | 3.89 |
| Body Mass Index (calculated) | 53 | 3.89 |
| McCabe Score at admission | 92 | 6.76 |
| Cirrhosis | 37 | 2.72 |
| Cancer | 29 | 2.13 |
| Immunodeficiency | 21 | 1.54 |
| Heart failure | 51 | 3.75 |
| Diabetes mellitus | 21 | 1.54 |
| Chronic respiratory failure | 25 | 1.84 |
| Chronic kidney disease under RRT | 16 | 1.18 |
| SAPS II | 11 | 0.81 |
| SOFA | 0 | 0 |
| Serum sodium (mmol/L) | 36 | 2.65 |
| Serum potassium (mmol/L) | 41 | 3.01 |
| Serum bicarbonate (mmol/L) | 84 | 6.17 |
| Serum creatinine (mg/dL) | 56 | 4.11 |
| PaO²/FiO² (mmHg) | 101 | 7.42 |
| Main cause of admission | 0 | 0 |
| Vasopressors at day 0 | 0 | 0 |
| RRTat day 0 | 0 | 0 |
| Diuresis (mL) | 5 | 0.37 |
| Serum urea (mg/dL) | 64 | 4.70 |
| Bilirubin (µmol/L) | 397 | 29.17 |
| Vital status at day 60 | 7 | 0.51 |

# Table S4. Effect of the exposure to the POINCARE-2 strategy on the cumulative number of MVFDs and VFDs

|  |  | **Complete cases^a^** | | | | | | | | | **Multiple imputation^b^** | | | | | | | | | |  |
| --- | --- | --- | --- | --- | --- | --- | --- | --- | --- | --- | --- | --- | --- | --- | --- | --- | --- | --- | --- | --- | --- |
|  |  | **ZI part^c^** | | | | | **Count part^d^** | | | | **ZI part^c^** | | | | | | **Count part^d^** | | | |  |
|  |  | Exp(parameter) | **95% CI** | | **p** | | Exp(parameter) | **95% CI** | **p** | Exp(parameter) | | **95% CI** | | | **p** | Exp(parameter) | | | **95% CI** | **p** | |
| MVFDs | without counfounders adjustment^1^ | 1.4 | | 1.002 - 2.04 | | 0.049 | 0.92 | 0.86 – 0.99 | 0.03 | NA | | | NA | NA | | NA | | NA | | | NA |
|  | adjusted on measured confounders^2^ | 1.3 | | 0.94 – 1.93 | | 0.11 | 0.92 | 0.85 – 0.98 | 0.02 | 1.3 | | | 0.99 – 1.79 | 0.06 | | 0.93 | | 0.88 – 0.99 | | | 0.02 |
| VFDs | without counfounders adjustment^1^ | 1.5 | | 0.94 - 2.51 | | 0.09 | 0.95 | 0.91 - 0.99 | 0.01 | NA | | | NA | NA | | NA | | NA | | | NA |
|  | adjusted on measured confounders^3^ | 1.1 | | 0.67 - 1.96 | | 0.61 | 0.98 | 0.94 - 1.02 | 0.33 | 1.2 | | | 0.78 - 1.95 | 0.34 | | 0.98 | | 0.94 - 1.01 | | | 0.22 |
| Models : | | | | | | | | | | | | | | | |  |  |  |  |  |  |
| 1 adjusted only on class variables: Center and Secular time  2 adjusted on Center, Secular time, Age (years), Main cause of admission, SOFA (~) | | | | | | | | | | | | | | | |  |  |  |  |  |  |
| 3 adjusted on Center, Secular time, Age (years), Heart failure, Chronic respiratory failure, Chronic kidney disease under RRT, McCabe Score at admission, SAPS II (*), Main cause of admission, Serum potassium (mmol/L), SOFA (~), Serum creatinine (mg/dL), RRT (#) at day 0 | | | | | | | | | | | | | | | |  |  |  |  |  |  |
| Analyses : | | | | | | | | | | | | | | | |  |  |  |  |  |  |
| a Observations with missing data are excluded (n=981) | | | | | | | | | | | | | | | |  |  |  |  |  |  |
| b Number of imputation datasets = 4 (n=1361) / NA for without confounders adjustment model because of non-missing data | | | | | | | | | | | | | | | |  |  |  |  |  |  |
| c Results of the zero-inflated part of the ZI-negbin models | | | | | | | | | | | | | | | |  |  |  |  |  |  |
| d Results of the count part of the ZI-negbin models | | | | | | | | | | | | | | | |  |  |  |  |  |  |
| * SAPS : Simplified Acute Phyisiology Score | | | | | | | | | | | | | | | |  |  |  |  |  |  |
| ~ SOFA : Sequential Organ Failure Assessment | | | | | | | | | | | | | | | |  |  |  |  |  |  |
| # RRT : Renal-replacement therapy | | | | | | | | | | | | | | | |  |  |  |  |  |  |

# Table S5. Effect of the exposure to the POINCARE-2 strategy on the occurrence of unexpected harmful events and renal damage

|  |  | **Complete cases^a^** | | | | **Multiple imputation^b^** | | |
| --- | --- | --- | --- | --- | --- | --- | --- | --- |
|  |  | **OR(Score > 75 %)** | **95% CI** | **p** | **OR(Score > 75 %)** | | **95% CI** | **p** |
| Unexpected harmful events | without counfounders adjustment^1^ | 1.4 | 0.97 - 2.08 | 0.071 | NA | | NA | NA |
|  | adjusted on measured confounders^2^ | 1.3 | 0.86 - 1.90 | 0.224 | 1.3 | | 0.94 - 1.77 | 0.120 |
| Renal damage | without counfounders adjustment^1^ | 1.2 | 0.82 - 1.72 | 0.364 | NA | | NA | NA |
|  | adjusted on measured confounders3 | 1.3 | 0.87 - 1.90 | 0.204 | 1.2 | | 0.89 - 1.72 | 0.195 |
| Models: | | | | | | | | |
| 1 adjusted only on class variables: Center and Secular time | | | | | | | | |
| 2 adjusted on Center, Secular time, McCabe Score at admission, SAPS II (*), Main cause of admission, Weight (kg), Serum potassium (mmol/L), SOFA (~) | | | | | | | | |
| 3 adjusted on Center, Secular time, McCabe Score at admission, SAPS II (*), Main cause of admission, Weight (kg), SOFA (~), Serum creatinine (mg/dL), RRT (#) at day 0 | | | | | | | | |
| Analyses : | | | | | | | | |
| a Observations with missing data are excluded (n=981) | | | | | | | | |
| b Number of imputation datasets = 4 (n=1361) / NA for without confounders adjustment model because of non-missing data | | | | | | | | |
| * SAPS: Simplified Acute Phyisiology Score | | | | | | | | |
| ~ SOFA: Sequential Organ Failure Assessment | | | | | | | | |
| # RRT : Renal-replacement therapy | | | | | | | | |

# Figure S1. POINCARE-2 strategy of fluid balance control in critically ill patients (reproduced from 9)


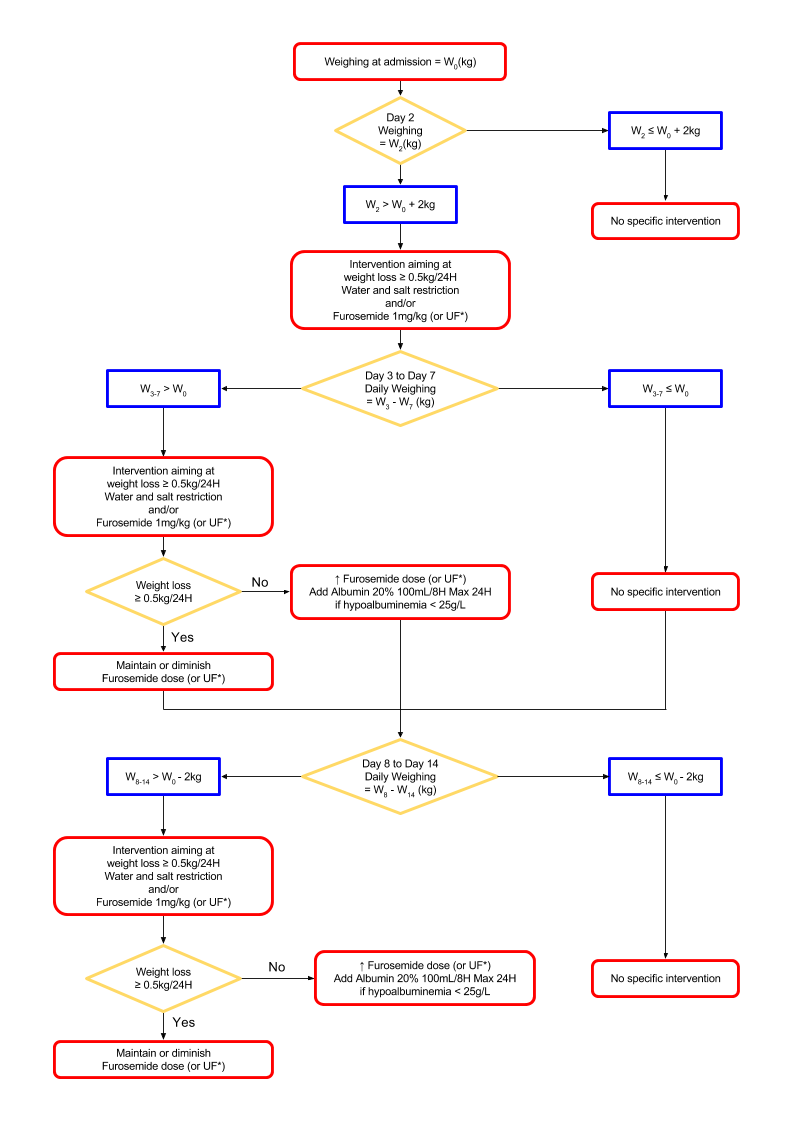


# Figure S2. Computation method for the score of exposure to the POINCARE-2 strategy


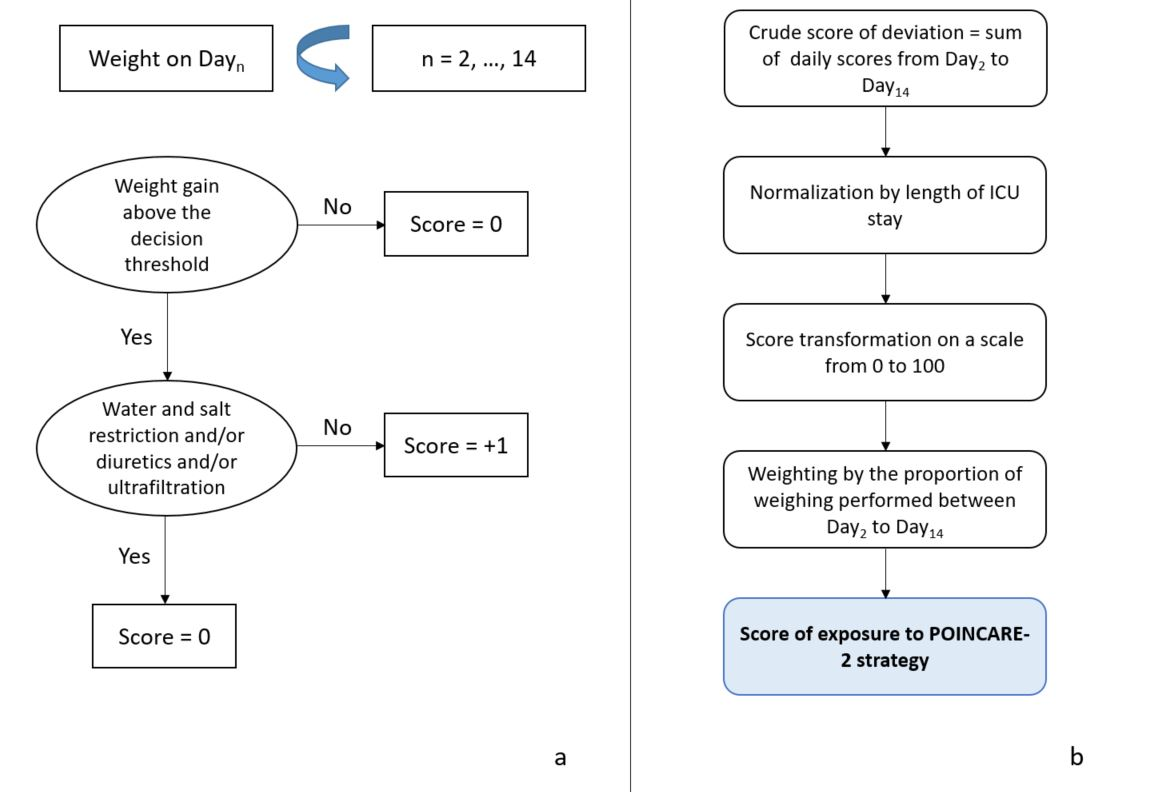

Supplement: Supplementary file 1 — Additional file 1: Supplementary tables and figures. [file 13054_2023_4701_MOESM1_ESM.docx]
